# Novel CMKLR1 Inhibitors for Application in Demyelinating Disease

**DOI:** 10.1038/s41598-019-43428-8

**Published:** 2019-05-09

**Authors:** Vineet Kumar, Melissa LaJevic, Mallesh Pandrala, Sam A. Jacobo, Sanjay V. Malhotra, Brian A. Zabel

**Affiliations:** 1 Department of Radiation Oncology, Stanford University, Palo Alto, California, USA; 2 Radiology, Stanford University, Palo Alto, California, USA; 3 Palo Alto Veterans Institute for Research, Veterans Affairs Palo Alto Health Care System, Palo Alto, California, USA

**Keywords:** Autoimmunity, Lead optimization

## Abstract

Small molecules that disrupt leukocyte trafficking have proven effective in treating patients with multiple sclerosis (MS). We previously reported that chemerin receptor chemokine-like receptor 1 (CMKLR1) is required for maximal clinical and histological experimental autoimmune encephalomyelitis (EAE); and identified CMKLR1 small molecule antagonist 2-(α-naphthoyl) ethyltrimethylammonium iodide (α-NETA) that significantly suppressed disease onset *in vivo*. Here we directly compared α-NETA versus FDA-approved MS drug Tecfidera for clinical efficacy in EAE; characterized key safety/toxicity parameters for α-NETA; identified structure-activity relationships among α-NETA domains and CMKLR1 inhibition; and evaluated improved α-NETA analogs for *in vivo* efficacy. α-NETA proved safe and superior to Tecfidera in suppressing clinical EAE. In addition, we discovered structurally differentiated α-NETA analogs (primarily ortho- or para-methoxy substitutions) with significantly improved target potency *in vitro* and improved efficacy *in vivo*. These findings suggest that α-NETA-based CMKLR1 inhibitors may prove safe and effective in treating demyelinating diseases and potentially other autoimmune disorders.

## Introduction

Multiple sclerosis (MS) is a disabling demyelinating disease of the central nervous system (CNS) that affects approximately 2.5 million people worldwide^1^. The pathomechanism of MS and its mouse-model counterpart experimental autoimmune encephalomyelitis (EAE) is based on inflammatory autoreactive leukocytes entering the CNS, destroying axonal myelin, and contributing to neurodegeneration^2^. FDA-approved disease modifying therapies such as dimethyl fumarate (Tecfidera) aim to prevent MS relapses and slow neurodegeneration, although the existing medications are only partially effective and can have undesirable side-effects (e.g. lymphopenia, increased susceptibility to opportunistic infections, progressive multifocal leukoencephalopathy)^3^. Even though a number of MS treatments are available, due to the heterogeneity of the MS disease process, individual patient responses, and medication toxicities, there remains a substantial unmet clinical need for improved therapies.

Small molecule therapeutics that target leukocyte trafficking pathways can reduce disease activity and improve clinical outcomes in MS. For example, FTY720 (Fingolimod, Gilenya), a small molecule that targets S1P receptors, is an approved treatment for MS that inhibits the migration of autoreactive T cells into the CNS by triggering lymphocyte sequestration in lymph nodes (reviewed in^4^). S1P receptors are expressed on most leukocytes, and thus agents that target S1P receptors may lead to systemic defects in immunity, and incidences of lymphoproliferative disorders have been reported^5–7^. Agents that selectively target the trafficking of key inflammatory cell subsets involved in the pathophysiology of MS may therefore be superior to current treatment strategies.

Chemokine-like receptor-1 (CMKLR1) is a chemoattractant receptor that binds chemerin, a proteolytically regulated leukocyte chemoattractant^8^. CMKLR1 is expressed by key effector cells in EAE and MS, including macrophages, subsets of dendritic cells (DC), natural killer (NK) cells and microglia^9–12^. Lande *et al*. reported chemerin co-localization with intralesional endothelial cells in the brains of MS patients, and identified CMKLR1+ leukocytes in the leptomeninges and in perivascular cuffs of chronic and active MS lesions^13^. In preclinical studies, we showed that CMKLR1-knockout (KO) mice develop less severe clinical and histological EAE than wild-type (WT) mice^12^. We sought to identify CMKLR1 inhibitors to pharmaceutically recapitulate the CMKLR1 KO phenotype in EAE. We identified a lead CMKLR1 antagonist α-NETA, which inhibited chemerin-driven CMKLR1 signaling (β-arrestin2 and chemotaxis) *in vitro* and suppressed EAE *in vivo*^14^. Here we investigated critical drug developability features of α-NETA, including *in vitro* and *in vivo* safety assessments; identified SAR among α-NETA domains and CMKLR1 inhibition; and used SAR-guided medicinal chemistry to generate α-NETA analogs with improved *in vitro* target potency and *in vivo* efficacy in suppressing EAE.

## Results

### α-NETA is superior to Tecfidera in suppressing clinical EAE *in vivo*

**Figure 1:**
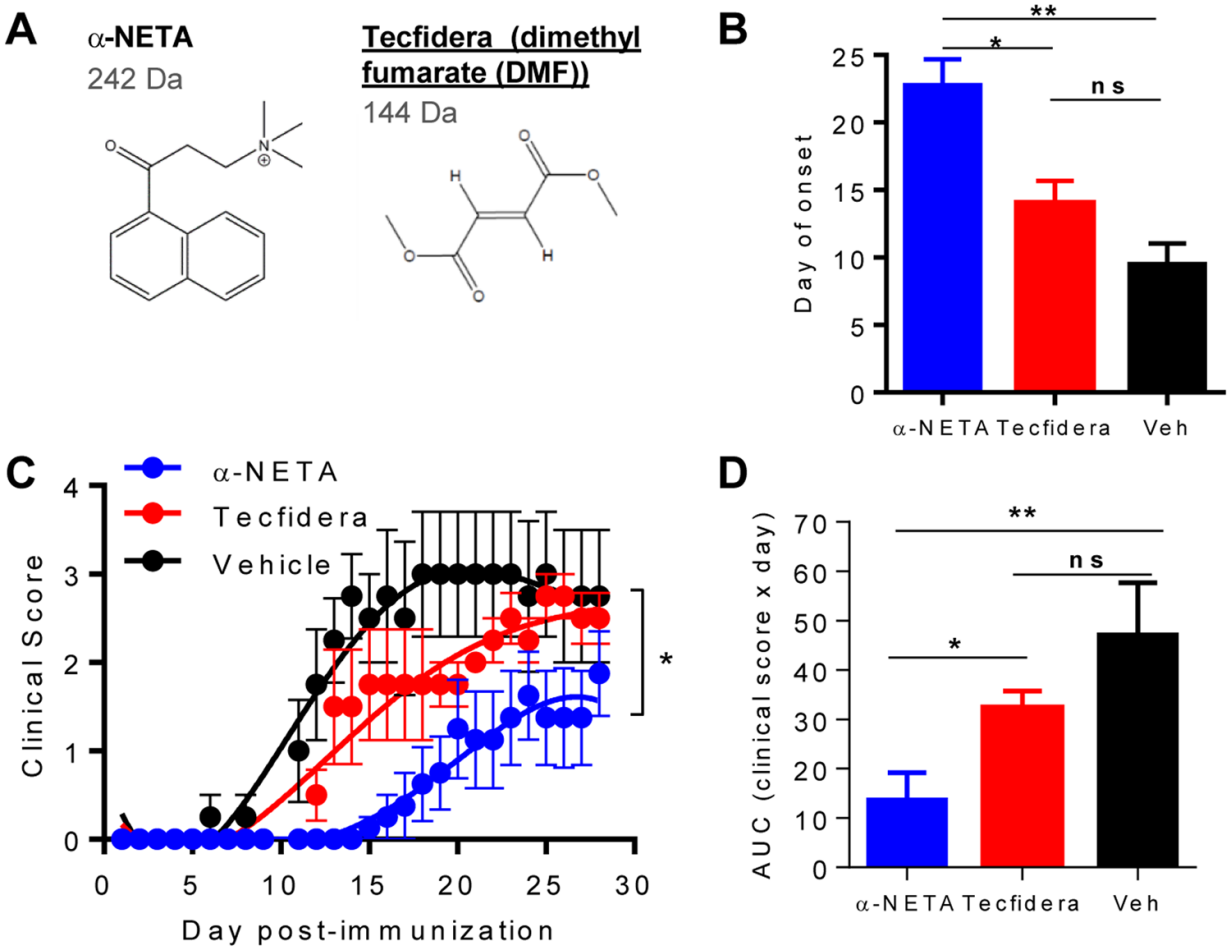
Comparing CMKLR1 antagonist α-NETA and FDA-approved Tecfidera (dimethyl fumarate, DMF) in suppressing experimental autoimmune encephalomyelitis (EAE). (**A**) Structure and features of α-NETA and DMF. (**B**) EAE was induced in C57BL/6 mice by active immunization with MOG_35–55_/CFA. Mice received α-NETA (10 mg/kg daily; n = 8 mice), DMF (10 mg/kg daily, n = 4 mice)), or vehicle control (10% captisol, n = 4 mice) beginning at the time of disease induction and were monitored daily for clinical disease as follows: 0, normal; 1, limp tail; 2, hind limb weakness; 3, hind limb paralysis; 4, forelimb and hind limb paralysis; 5, dead. Day of onset of clinical signs (assumes worst case scenario d29 onset for 2 mice in the α-NETA group). Mean + SEM, *p < 0.05, **p < 0.01 by one-way ANOVA. ns, not significant. (**C**) Mean clinical score - SEM. *p < 0.0001 by F-test (extra sum of squares) comparing the shapes of the 4^th^ order polynomial curves for each treatment group, rejecting the null hypothesis that one curve fits all data sets. (**D**) The integrated clinical score over time for each animal was calculated as area under the curve (AUC). Mean + SEM, *p < 0.05, **p < 0.01 by one-way ANOVA.

We compared α-NETA vs. FDA-approved Tecfidera (Fig. 1A) for efficacy in suppressing clinical signs of EAE induced by immunization with myelin oligodendrocyte glycoprotein peptide 35–55. When administered daily at the same dose (10 mg/kg) in the same vehicle (10% captisol in water), α-NETA significantly delayed disease onset compared with either Tecfidera or vehicle control (day of EAE onset for α-NETA: 21 ± 2; Tecfidera: day 14 ± 2; vehicle: day 10 ± 2, mean ± SEM, *p < 0.05 by ANOVA; Fig. 1B). The severity of clinical EAE was also significantly suppressed by α-NETA compared with Tecfidera or vehicle control (Fig. 1C). In addition, we previously showed that α-NETA treatment significantly reduced mononuclear cell infiltrates within the CNS^14^. We also quantified the total disease experienced by animals in the three treatment groups by area under the curve analysis (Fig. 1D). Thus, by these methods of analyses of clinical EAE, α-NETA-treated animals experienced significantly less severe disease than Tecfidera or vehicle-treated controls (Fig. 1).

### *In vitro* safety analysis of α-NETA

**Figure 2:**
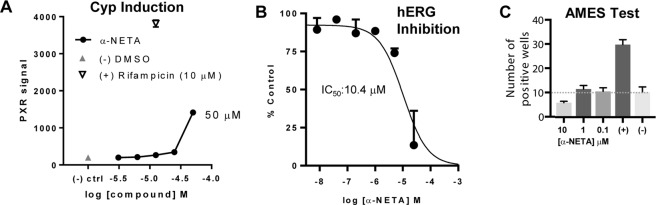
*In vitro* safety assessment. (**A**) Cyp induction. Pregnane X receptor (PXR) activation was used as a marker of Cyp induction. An engineered human hepatoma cell line with a PXR luciferase reporter was incubated with the indicated concentrations of α-NETA, Rifampicin (positive control), or DMSO (negative control), and luciferase activity assessed. Mean ± range of duplicate wells. (**B**) hERG inhibition. Patch-clamp assay with single CHO-hERG cell transfectants were used to quantify potential α-NETA-dependent hERG inhibition (2–3 cells per compound concentration). Basal hERG current was measured, α-NETA (0.008, 0.04, 0.2, 1, 5, 25 uM) was added, the cell was depolarized, the hERG tail current was measured, and IC50 determined. Mean + range or SEM displayed. (**C**) Ames test for genotoxicity. Histidine revertants were quantified following exposure to α-NETA (0.1, 1, 10 uM; 48 wells/dose). Mean number of positive wells + SEM displayed. (−) control: PBS; (+) control: sodium azide.

We assessed potential off-target activity of α-NETA in inhibiting or inducing the activity of cytochrome P450 (Cyp) drug metabolizing enzymes. Effects on the Cyp enzymes are important in avoiding potentially serious drug-drug interactions that can derail drug development efforts^15^. In human liver microsomal Cyp activity assays, α-NETA had little inhibitory activity against Cyp1A2, 2C9, 2C19, 2D6, and 3A4, which are the main drug detoxifying enzymes in the Cyp family (Table 1). α-NETA had some inhibitory activity against Cyp2C8 (IC50: 1.5 uM) and was a relatively potent inhibitor of Cyp2B6 (IC50: 0.12 uM). No time-dependent (mechanism-based) Cyp inhibition was detected (not shown). To assess possible induction of Cyp enzymes, we used a reporter cell line to assess activation of nuclear receptor PXR, which is commonly induced by Cyp enzymes. α-NETA did not induce substantial PXR activity at concentrations up to 50 uM (Fig. 2A).

**Table 1:** Cyp Inhibition.

Isoform	IC50 (uM)
Cyp1A2	8^a^
Cyp2B6	0.12^b^
Cyp2C8	1.5^b^
Cyp2C9	14.6^b^
Cyp2C19	84^b^
Cyp2D6	>10^c^
Cyp3A4	7.5^b^

^a^PubChem BioAssay ID 1476.

^b^Human liver microsomes were pre-incubated with α-NETA (0.1–100 mM, duplicate wells). IC50s values were calculated following incubation with cyp-specific substrates.

^c^PubChem BioAssay ID 1461.

Cardiotoxicity by off-target interference with human Ether-a-go-go Related Gene (hERG) potassium channels is a major safety concern in drug development^16^. In preliminary studies, we assessed potential off-target hERG inhibition by α-NETA using a gold-standard patch-clamp single cell depolarization assay. α-NETA had little inhibitory activity against hERG (IC_50_ >10 uM) (Fig. 2B).

Genotoxicity by off-target mutagenic effects represents an important safety concern^17^. In preliminary studies, we assessed potential off-target genotoxicity by α-NETA using the Ames test. α-NETA did not induce mutagenesis (monitored by reversion of an obligate histidine mutation) at concentrations up to at least 10 uM (Fig. 2C).

### *In vivo* toxicity analysis for α-NETA

**Figure 3:**
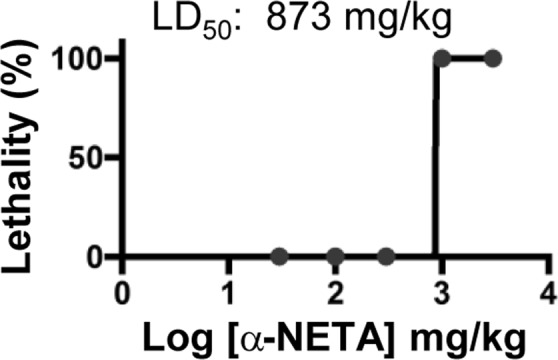
Acute single dose toxicity (LD50) of α-NETA. WT C57BL6 mice were treated with the following doses of α-NETA: 3000, 1000, 300, 100, 30, 0 mg/kg by oral gavage (200 ul/dose in 10% captisol). Lethal effects were observed within hours for the two highest doses. The remaining mice were monitored for 14 days. Graph depicts lethality at each dose, n = 3 per mice/dose.

**Figure 4:**
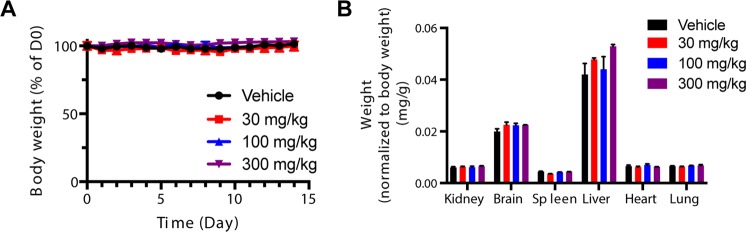
*In vivo* safety: Repeat dosing of α-NETA does not affect body weight or vital organ weight/gross morphology. WT C567/BL6 mice were treated with various doses of α-NETA (300, 100, 30, 0 mg/kg) by oral gavage (in 10% captisol vehicle) daily for 14 days. (**A**) Mouse body weight was recorded daily and displayed as percent initial weight on d0, mean + SEM, n = 3 mice per dose. (**B**) On day 14, the mice were euthanized and the wet weight of the indicated organs determined and displayed, normalized to body weight. Mean weight + SEM, n = 3 mice/dose. No significant differences noted.

We assessed the acute single dose toxicity (LD_50_) of α-NETA (p.o.). The calculated LD_50_ was 873 mg/kg, (Fig. 3). Lethality was an off-target effect, as CMKLR1 KO mice also died following 1000 and 3000 mg/kg dosing (not shown). In repeat dosing safety studies, α-NETA administered for 14 days p.o. at up to 300 mg/kg/day had no effect on body weight (Fig. 4A) or the wet weight or gross morphological appearance of vital organs (Fig. 4B). Thus, the no-observable-adverse-effect-level (NOAEL) level for α-NETA in a repeated dosing regimen for up to 14 days is at least 300 mg/kg.

### Structure-activity relationship (SAR)

**Figure 5:**
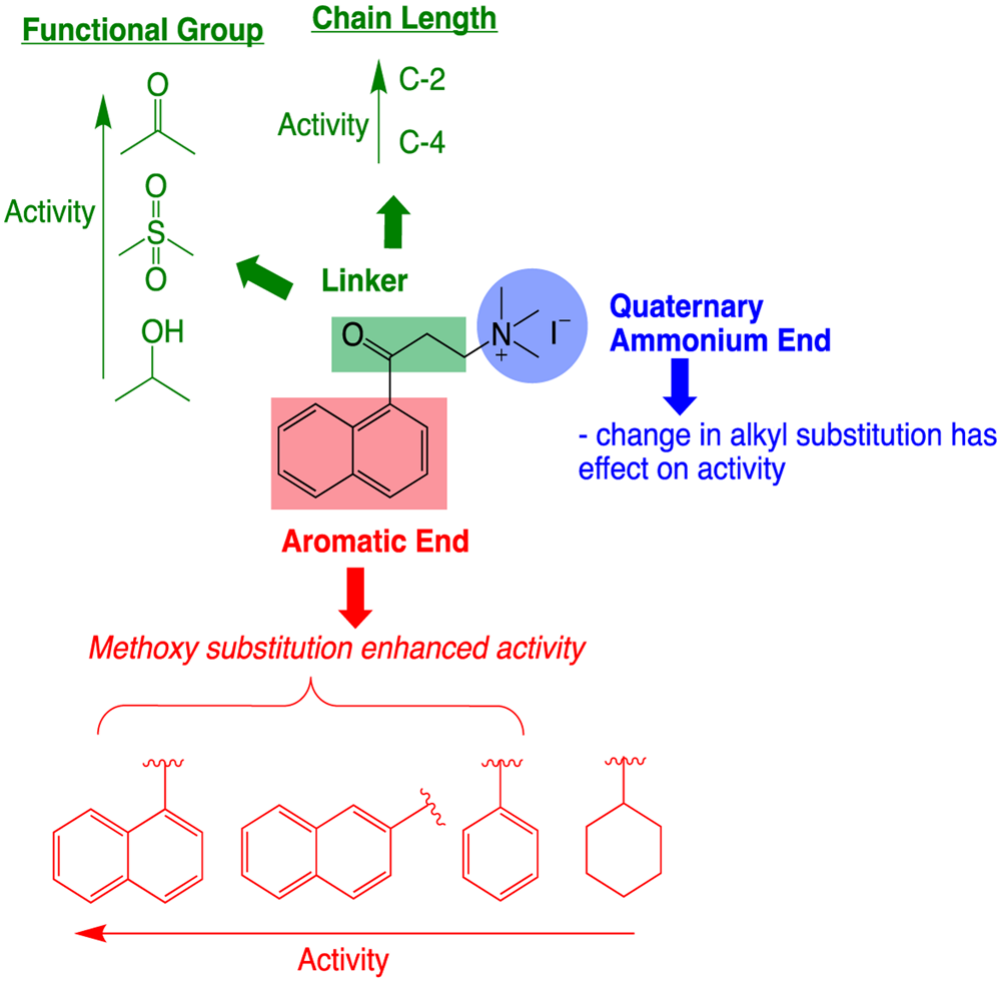
SAR of α-NETA analogs for CMKLR1 inhibition. Thin arrows indicate structural features leading to increased potency.

α-NETA consists of: i) a quaternary ammonium end, ii) an aromatic end consisting of α-naphthyl group, and iii) a three-carbon linker with a carbonyl functional group (Fig. 5). We synthesized analogues of α-NETA by systematic modifications at these three sites to study the structure-activity relationship (SAR) using the β-arrestin2 assay.

**Figure 6:**
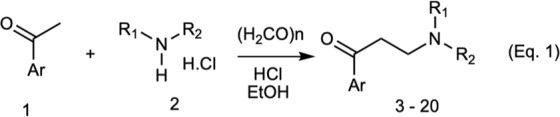
Synthesis of tertiary amine α-NETA analogues with modified ammonium and aromatic groups.

In the first series of modifications we kept the linker as it is and modified the quaternary ammonium and aromatic ends of α-NETA. We synthesized a set of tertiary amines with varied N-alkyl substitutions and different aromatic rings. These compounds were synthesized from corresponding ketones **1** and hydrochloride salts of dialkylamines **2** using mannich reaction (Fig. 6, Eq. 1) and screened for their activity using β-arrestin2 assay (Table 2). In case of α-naphthyl derivatives (compounds **3**–**5**), β-arrestin2 inhibition activity increases with bulkier substitution on nitrogen. Similar structural-activity trend was also observed with phenyl derivatives (compounds**12** and **13**). In contrast, activity decreases with bulkier N-substitution in α-naphthyl derivatives. Effect of methoxy substitution at appropriate position also had prominent effect on the activity, as suggested by lower IC_50_ values for compounds **6** and **19**. The inhibitory effect of compound **6** on chemerin-dependent β-arrestin2 signaling was not significantly different from the effect of α-NETA (p > 0.05 by two-tailed t-test) (Table 2). Replacing naphthyl group with cyclohexyl (compound **20**) resulted in complete loss of activity (Table 2).

**Table 2:** SAR of tertiary amines on CMKLR1 inhibition.

Cmpd ID	Ar	R1	R2	β-arrestin IC_50_ µM mean ± SEM (n)
α-NETA				4.9 ± 1.5 (18)
**3**		Me	Me	18.8 (1)
**4**		Me	Et	17.9 (1)
**5**		Et	Et	8.9 ± 2.4 (4)
**6**		Me	Me	6.2 ± 2.5 (3) n.s.
**7**		Me	Me	18.5 (1)
**8**		Et	Et	71.3 (1)
**9**		Me	Et	39.6 (1)
**10**		Me	Bu	44.5 (1)
**11**		Me	Me	>100 (2)
**12**		Me	Me	>100 (1)
**13**		Me	Et	16.8 ± 8.3 (3)
**14**		Et	Et	77.8 (1)
**15**		Me	Bu	>100 (4)
**16**		Me	Me	>100 (1)
**17**		Me	Me	>100 (1)
**18**		Me	Me	64 (1)
19		Me	Me	10.3 ± 4.5 (4)
20		Me	Me	>100 (1)

n.s., not significant by two-tailed t-test vs. α-NETA.

**Figure 7:**
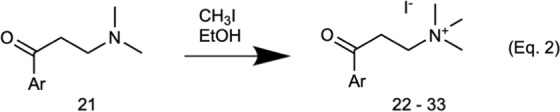
Synthesis of quaternary amine α-NETA analogues with modified aromatic groups.

Next, we evaluated the effect of change in aromatic end on the β-arrestin2 activity, keeping quaternary ammonium end and linker unchanged (similar to α-NETA). Corresponding tertiary amines **21** were quaternized using methyl iodide to obtain desired compounds **22**–**33** (Fig. 7, Eq. 2). β-arrestin2 assay showed a trend in the activity of these compounds decreasing in the following order: α-naphthyl (**22**, IC_50_ 2.3 µM) > β-naphthyl (**23**, IC_50_ 3.1 µM) >phenyl (**24**, IC_50_ 4.3 µM) >cyclohexyl (**25**, IC_50_ 11.9 µM) (Table 3). Interestingly, methoxy substitution on all the aromatic rings increased the potency. In general, *ortho* or *para* methoxy compounds (**26**, **27** and **30**) showed higher β-arrestin2 activity in comparison to *meta* substituted derivatives (compounds **28** and **31**). A secondary chemotaxis assay was also performed on selected hit compounds, which also showed higher potency for methoxy-substituted compounds (Table 3). Surprisingly, phenyl derivative **24** showed activity in β-arrestin2 assay but did not show any activity in chemotaxis assay, while the methoxy-substituted phenyl derivatives **26**, **27** and **30** were highly active in both assays (Table 3). While the inhibitory effect of compound **27** on chemerin-dependent β-arrestin2 signaling was not significantly different from α-NETA (p > 0.05 by two-tailed t-test), compound **27** inhibited chemerin-dependent cell migration with significantly better potency than α-NETA (p < 0.05 by two-tailed t-test) (Table 3).

**Table 3:** SAR of aryl group on CMKLR1 inhibition.

Cmpd ID	Ar	β-arrestin IC_50_ µM mean ± SEM (n)	Chemotaxis IC_50_ µM mean ± SEM (n)
**22**(α-NETA)		4.9 ± 1.5 (18)	37.0 ± 8.9 (6)
**23**		2.5 ± 0.9 (5)	24.1 (1)
**24**		5.6 ± 0.7 (6)	>100 (1)
**25**		11.9 (1)	>100 (1)
**26**		2.9 ± 1.6 (5)	7.2 (1)
**27**		1.9 ± 0.2 (8) n.s.	4.5 ± 1.5 (3)*
**28**		3.8 ± 1.7 (4)	14.6 ± 4.8 (2)
**29**		4.8 ± 1.6 (8)	17.4 ± 2.2 (4)
**30**		2.1 ± 1.1 (7)	6.8 (1)
**31**		6.0 ± 2.2 (6)	>100 (4)
**32**		1.5 ± 0.3 (3)	20 ± 6.2 (3)
**33**		0.6 ± 0.3 (3)	10.7 (1)

n.s., not significant by two-tailed t-test vs. α-NETA.

*p < 0.05 by two-tailed t-test vs. α-NETA.

**Figure 8:**
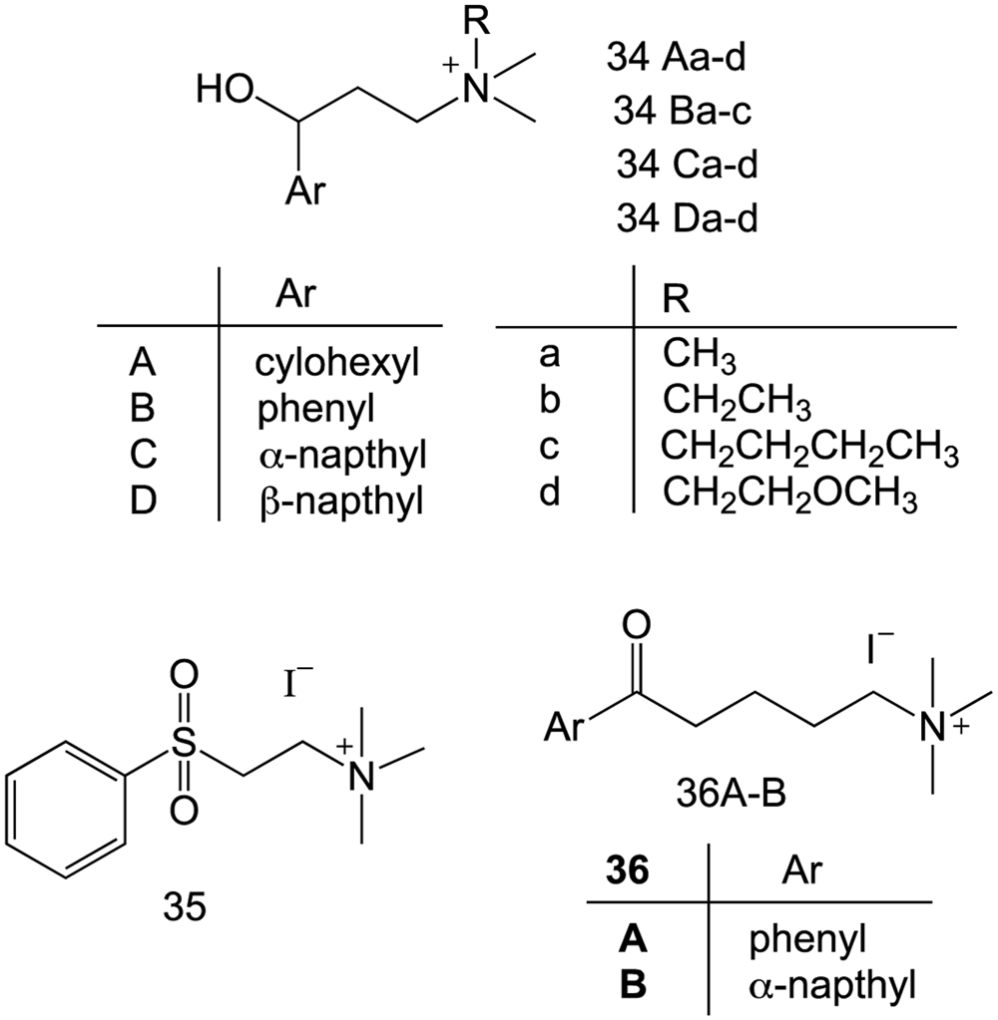
Structures of α-NETA analogues with modified linker functional groups and chain length.

To further expand the SAR, the carbonyl functional group of the linker moiety of α-NETA derivatives was modified to hydroxyl and sulfone groups to obtain corresponding functionalized -quaternary ammonium salts **34A-D** and **35**, respectively (Fig. 8). However, all these compounds with modified functional group were inactive in β-arrestin2 assay. We also modified alkyl chain length of the linker from C-2 to C-4 carbons to obtain compounds **36A-B** (Fig. 8). These compounds with elongated linker chain length were also inactive in β-arrestin2 assay.

Finally, we asked if new compounds generated via our preliminary SAR studies were effective in suppressing EAE. We were particularly interested to see if any of the new, more potent compounds were similarly more efficacious than α-NETA *in vivo*. We selected the comparatively more potent methoxy substituted derivative of α-NETA **27** and its tertiary amine counterpart **6** for *in vivo* testing. While **6** was similarly effective in suppressing the integrated clinical score as α-NETA, **27** proved to be superior to α-NETA in blunting severe EAE disease: both the maximum clinical score and the incidence of hind limb paralysis were significantly reduced by **27** vs. α-NETA (Table 4).

**Table 4:** α-NETA and analogs 27 and 6 suppress clinical EAE.

Treatment Group	AUC (Mean ± SEM)	Day of onset (Mean ± SEM)	Maximum Score (Mean ± SEM)	Incidence of hind limb paralysis
Vehicle	44.7 ± 5.5	12.5 ± 0.7	2.9 ± 0.1	7/8 (87.5%)
α-NETA	31.9 ± 4.0*	14.4 ± 0.6*	2.6 ± 0.2	5/8 (62.5%)
27	24.3 ± 4.2*	16.0 ± 0.9*	2.1 ± 0.1^¶^	1/8 (12.5%)^§^
6	25.2 ± 4.0*	14.5 ± 0.4*	2.4 ± 0.2	3/8 (37.5%)

EAE was induced in WT C57/BL6 mice by active immunization with MOG_35–55_/CFA/PTX and monitored daily for clinical signs as previously described. Mice were treated with α-NETA, 6, 27 (10 mg/kg), or vehicle (10% captisol) daily, beginning at the time of disease induction and ending on D21. n = 8 mice per group. AUC = area under the curve, calculated for each individual mouse as mean clinical score x day.

*p < 0.05, as determined one-way ANOVA compared to vehicle.

^¶^p < 0.05 as determined by t-test compared to α-NETA.

^§^p < 0.05 as determined by Chi-square (2 × 2 contingency table) test compared to α-NETA.

## Discussion

In this study, we investigated key efficacy, safety, and SAR features of CMKLR1 antagonist α-NETA. In terms of efficacy, α-NETA outperformed Tecfidera in suppressing clinical signs of EAE. With respect to safety, we did not identify any major liability for α-NETA in the industry-standard *in vitro* or *in vivo* toxicity studies investigated in this report. Through rationale medicinal chemistry modifications to α-NETA structure, we identified specific SAR among α-NETA domains and CMKLR1 inhibition. Importantly, we discovered new α-NETA analogs with improved CMKLR1 target potency, one of which proved superior to α-NETA in suppressing severe clinical EAE *in vivo*. Thus, α-NETA-based CMKLR1 small molecule antagonists offer a promising, developable approach to treat MS in the clinic.

Although Tecfidera was approved by the FDA for the treatment of MS in 2013, dimethyl fumarate and its metabolites have a long, 60-year history as an effective treatment for autoimmune psoriasis^18^. Dimethyl fumarate has myriad biological effects when administered *in vivo*, making it difficult to define the cellular and molecular mechanisms underlying its efficacy. Tecfidera is thought to promote protective anti-oxidant activities via activation of nuclear factor erythroid-2-related factor (NRF2)^19^; and to modulate immune responses to both favor immune suppression and to restrict leukocyte infiltration into the CNS, possibly via agonizing hydroxycarboxylic acid receptor 2 (HCAR2)^19,20^. *In vitro* cell model studies and *in vivo* rodent studies support that dimethyl fumarate protects neurons from oxidative damage via activation/translocation of NRF2 to the nucleus, where it transcribes several anti-apoptotic, neuroprotective, and anti-oxidant genes (e.g. heme oxygenase-1 (HO-1), NAD(P)H quinone dehydrogenase 1 (NQO1), GSTP1 (others glutathione-S-transferase), superoxide dismutase-2 (SOD2), Sulfiredoxin-1 (SRXN1), and ferritin heavy chain 1 (FTH1)^19^. In MS patients, Tecfidera alters the composition of peripheral blood leukocytes to promote anti-inflammatory conditions, reducing absolute numbers and percentages of CD8+ T cells, CD45RO+CD4+ memory T cells, Th1 (CXCR3+) and Th17 (CCR6+) CD4+ T cells, memory B cells, and CD16+ CD56low NK cells; and increasing percentages of CD45RA+CD4+ naïve T cells, Th2 (CCR3+) CD4+ T cells, regulatory B cells, and CD16loCD56bright NK cells^19^. In rodent EAE models, Tecfidera reduces leukocyte infiltration into the CNS, which correlates with preclinical efficacy^20^. The aggregate effect of these diverse biological changes in many MS patients is clinical improvement.

The mechanism of action for α-NETA in suppressing EAE is consistent with inhibition of leukocyte infiltration into the CNS^14^. Several recent studies also support a specific role for α-NETA in inhibiting chemerin/CMKLR1-associated functions *in vivo*: α-NETA blocked the recruitment of CMKLR1 + DC into the CNS in EAE mice^21^. α-NETA inhibited certain adipokine functions of chemerin mediated by CMKLR1, suppressing white fat deposition and liver steatosis induced by high fat diet challenge *in vivo*^22^. In a germinal matrix hemorrhage brain injury model in neonatal rats, α-NETA reversed the protective effects of exogenous chemerin acting on CMKLR1+ microglia^23^. [Interesting, in this model chemerin increased *Nrf2* expression, while α-NETA reversed this effect^23^. It would be interesting to determine if α-NETA affects *Nrf2* expression/function in the CNS in EAE, as this may provide rationale for combination therapy with Tecfidera]. α-NETA reduced the clonogenicity and viability of CMKLR1+ neuroblastoma cell lines *in vitro*, and reduced tumor growth *in vivo* in a preclinical neuroblastoma model^24^. [In most cancers, higher chemerin expression by tumor tissue correlates with improved survival outcomes, likely due to increased anti-tumor leukocyte recruitment and surveillance^25,26^. Yet in neuroblastoma, the reverse correlation was reported, where chemerin seems to play an oncogenic role^24^]. Despite multiple examples supporting a specific role for α-NETA in interfering with chemerin/CMKLR1-dependent processes, it is possible that α-NETA, similar to Tecfidera, interacts with multiple molecular targets when administered *in vivo* that may or may not relate to its efficacy in EAE. For example, α-NETA was reported to inhibit choline acetyltransferase^27,28^; inhibit aldehyde dehydrogenase 1 family, member A1 (ALDH1A1)^14^; and agonize trace amine-associated receptor 5 (TAAR5)^29^. To what extent α-NETA interacts with these targets *in vivo*, and how that may impact EAE progression, remains unknown.

In our EAE studies, the efficacy of Tecfidera in inhibiting clinical disease is not as striking as in previously published reports^20,30^. Differences in our experimental design include the dose used for treatment, the drug vehicle (10% captisol in water), and the route of administration (s.c.), all of which were chosen to directly compare with α-NETA. Tecfidera has a dose dependent response in the MOG EAE model^31,32^, and an effective dose for limiting clinical score is inconsistent amongst published reports. In some studies, 20 mg/kg Tecfidera was not efficacious^33^; in others, 10 mg/kg daily dosing led to small but significant improvements in clinical scores^31^. Daily doses of 200 mg/kg significantly suppressed clinical EAE, but these doses exceed by 2–3-fold the relevant clinical doses for MS patients^32^. In the absence of pharmacokinetic data for α-NETA, we elected to dose both α-NETA and Tecfidera at 10 mg/kg daily based on our previous work, which showed significant efficacy for α-NETA in attenuating EAE without obvious adverse events^14^.

The Food and Drug Administration (FDA) requires extensive preclinical *in vitro* and *in vivo* safety testing as part of an Investigative New Drug filing. In agreement with data deposited in PubChem (NCBI), our results indicate that α-NETA had little effect on hERG potassium channel-driven action potentials, and thus reduced predicted risk of cardiotoxicity *in vivo* (Fig. 2B). With respect to Cyp enzymes, α-NETA inhibited Cyp2B6 (IC50: 0.12 μM) and Cyp2C8 (1.5 μM) (Table 1). Cyp inhibition does not necessarily derail drug development (e.g. there are FDA-approved drugs (Orphenadrine, Gemfibrozil), that inhibit Cyp2B6 and Cyp2C8, respectively). However, use of Cyp inhibitors, particularly in combination with other drugs, requires additional patient monitoring to avoid adverse events related to drug-drug-interactions. While there were some minor differences in Cyp enzyme inhibition IC_50_ values between publicly indexed PubChem data and our direct experimental data, there was a sizable discrepancy for Cyp3A4: we reported weak inhibition (IC_50_: 7.4 μM), while PubChem listed the IC_50_ as 0.2 μM^14^. It is possible that the use of different substrates (Midazolam in our study, vs. luciferin-6′ phenylpiperazinylyl in the PubChem-reported assay) could affect the IC_50_ value. We therefore attempted the assay with testosterone as a third additional substrate. The IC_50_ value in this case was 6.9 μM, in-line with our Midazolam results. Thus, for substrates that may be encountered *in vivo*, α-NETA is a weak Cyp3A4 inhibitor. In terms of *in vivo* safety assessment, our repeated dosing study in mice indicated that α-NETA was well-tolerated at doses up to 300 mg/kg p.o. for at least 14 days. Furthermore, the calculated single dose LD_50_ for α-NETA was 873 mg/kg, which by comparison is safer than caffeine (LD_50_ 367 mg/kg)^34^. A recent study reported that direct α-NETA injection (5 μg) into the uterine horn on days 6, 9 and 12 following fertilization resulted in significant embryo resorption, thus potentially contraindicating α-NETA for treatment during pregnancy^35^.

In our initial studies, we used a cell-based chemerin-dependent signaling assay (β-arrestin2 inhibition) to identify α-NETA as a CMKLR1 inhibitor with an IC_50_ value of 0.38 μM^14^. In the SAR studies reported here, we again used β-arrestin2 inhibition assay, and in all cases compared the activity of newly generated compounds versus contemporaneously-tested α-NETA. Here it is worth mentioning that α-NETA obtained from different sources gave varied results. Therefore, to maintain consistency all compounds were prepared in our lab and characterized for purity (>98%) prior to any biological testing. It is known that live cell-based assays that quantify inhibitory signals are more sensitive to such variables than enzymatic or ligand binding assays. As a reference for comparison, Bentz *et al*. compared the interlaboratory variability of IC_50_ values for 16 different inhibitors using a cell-based assay across 23 different research groups. The minimum difference in IC_50_ values (e.g. the most consistent data) was 20-fold between the lowest and highest IC_50_ values, while the maximum difference (e.g. the least consistent data) was 796-fold^36^. While our experiments were conducted in the same lab, the studies were temporally separated by 4–5 years using different sources of α-NETA, and thus some differences in IC_50_ values are expected. For SAR studies, contemporaneous comparison vs. parental α-NETA (and producing all compounds in-house) is crucial to identify improved-potency α-NETA-derivatives.

Overall SAR of our newly synthesized α-NETA analogues is summarized in Fig. 5. We made simple changes to modify three key sites of α -NETA to evaluate SAR, i.e. changing of ammonium head group, modification of length and functionality of the linker and changing the aromatic ring. Our studies indicated that the quaternary ammonium moiety is crucial for activity. Corresponding tertiary amines were comparatively less active in our assay. Any modification of the linker was also not tolerated. Compounds with modified functional groups or chain length did not show any activity. Changing the α-naphthyl to phenyl or a cyclohexyl ring reduced the activity, however, methoxy substitution at the aromatic rings significantly enhanced the β-arrestin2 activity (Fig. 5). These studies indicate that the aromatic ring of α -NETA is the only site where changes are tolerated. In our future studies we plan to generate more diversified α -NETA analogues by making complex changes at the aromatic ring site by incorporating heterocycles and substitutes aromatics.

In conclusion, our data suggests that α-NETA is well-tolerated *in vivo* at efficacious and supra-efficacious doses, has a reasonable *in vitro* safety profile with respect to parameters commonly applied to early stage drug development programs, and is effective in suppressing demyelinating disease as compared to Tecfidera. Furthermore, we successfully identified SAR among α-NETA domains and CMKLR1 inhibition and discovered methoxy-substituted derivatives with enhanced potency *in vitro* and enhanced efficacy *in vivo*. Thus, CMKLR1 antagonist α-NETA provides a strong base molecule to use as a benchmark for the development of improved α-NETA derivatives for the treatment of MS and potentially other autoimmune or inflammatory disorders.

## Methods

### Mice

C57BL/6 mice were purchased from The Jackson Laboratory. Female mice (8–12 weeks old) were used in all experiments. Animal experiments were conducted in accordance with approved Veterans Affairs, National Institutes of Health, and Institutional Animal Care and Use Committee guidelines.

### Cell culture

(hu)CMKLR1 transfectants (generated in L1.2 pre-B cell lymphoma cells) were grown in RPMI 1640 (Corning) supplemented with L-glutamine, penicillin-streptomycin, and 10% BCS and G418-sulfate (KSE Scientific). DiscoverX PathHunter® CHO-K1 CMKLR1 β-Arrestin Cells were grown in RPMI lacking phenol red (Gibco, Life Technologies), supplemented with L-glutamine, penicillin-streptomycin, sodium pyruvate, non-essential amino acid, BCS, hygromycin, and G418-sulfate.

### β-arrestin recruitment assay

CMKLR1-CHO-β-Arrestin cells were seeded in 96 well plates at 1 × 10^5^ cell/ml- 100 ul/well and incubated overnight at 37 °C, 5% CO_2_. The next day, media was removed and cells were pre-incubated in PBS with α-NETA or test compound (95 ul/well) in a 6 point dose response curve from 100 μM to 0.3 μM for 10 min at RT. (α-NETA and test compounds were reconstituted in DMSO; final DMSO concentration in each well was 0.1%). After 10 min, 5 ul of recombinant human chemerin (R&D Systems) at a final concentration of 20 nM was added to each well. Plates were incubated for 90 min at 37 °C, 5% CO_2_. Media was then removed and 50 ul of chemiluminescent substrate (Tropix Gal-Screen, Applied Biosciences) was added. The plate was incubated for 1 hr at RT and luminescence was detected using the SpectraMax M5 plate reader (Molecular Devices). For each independent trial (single replicate dose curves), data was normalized to the maximum signal per compound tested and an IC50 value was generated using GraphPad Prism software. The average IC50 value for n independent trials (as specified in the table) is reported.

### Chemotaxis assay

(hu)CMKLR1 L1.2 cells at 1 × 10^6^ cell/ml were treated with 5 mM sodium butyrate (Sigma) overnight to induce hCMKLR1 expression. The next day, cells were washed in migration media (RPMI + 0.5%BSA) and resuspended at 1.5–3 × 10^6^ cell/ml. Cells were pre-incubated at RT for 10 min with various concentrations of α-NETA or test compounds (α-NETA and test compounds were reconstituted in DMSO; final DMSO concentration in each well was 0.1%). 100 ul of cells were added to the upper chamber of a 24-well transwell plate (5 um pore size, Costar). Cells were allowed to migrate toward huChemerin (0.6 mM in migration media, bottom chamber) for 2 h, 37 °C, 5% CO_2_. The number of migrated cells was quantified by flow cytometry (BD LSR2), and live cells were distinguished based on FSC × SSC properties. The background for this assay was typically low with 0 cells migrating to the buffer alone. For each independent trial, data was normalized to the maximum migration per compound tested and an IC50 value was generated using GraphPad Prism software. The average IC50 value for n independent trials (as specified in the table) is reported.

### EAE

C57/BL6 mice were immunized (s.c. injection near inguinal LNs- 50 ul per side) with 100 ul/mouse of a 1:1 emulsion of Complete Freund’s Adjuvant (CFA): Myelin oligodendrocyte glycoprotein (MOG) peptide amino acids 35–35 (MEVGWYRSPFSRVVHLYRNGK), (MOG_35–55_, *Stanford Protein and Nucleic Acid Facility, Stanford, CA*). CFA consisted of 5 mg/ml mTB (Mycobacterium-tuberculosis H37 Ra, BD Difco) in IFA (Incomplete Freund’s Adjuvant, Sigma). MOG_35–55_ was reconstituted at 2 mg/ml in PBS. Mice also received 200 ng pertussis toxin (List Biological Laboratories, Inc) by i.p. injection at the time of immunization and two days later. Mice were randomly assigned treatment groups and received daily treatments of α-NETA, DMF, compound: 27 or 6, (10 mg/kg in 10% captisol) or vehicle (10% captisol in water) via s.c. injection on the flank beginning at the time of disease induction and ending on day 21–27. The dose of α-NETA was chosen based on our previous work^14^, which was efficacious and well-tolerated. For a direct comparison to α-NETA, the DMF and compound doses were also 10 mg/kg in 10% captisol. Mice were monitored daily for clinical disease and scored (0 = normal/healthy, 1 = limp tail, 2 = hindlimb weakness, 3 = hindlimb paralysis, 4 = hindlimb and forelimb paralysis, 5 = moribund/dead).

### α-NETA acute toxicity

C57/BL6 mice received one treatment of α-NETA (formulated in 10% captisol, Cydex Pharmaceuticals) at the following doses: 3000, 1000, 300, 100, 30, 0 mg/kg by oral gavage (200 ul/dose, 3 mice/group). Mouse survival was monitored and body weight was measured daily for 14 days. On day 14 the mice were euthanized and the wet weight for the following tissues: kidney, brain, spleen, liver, heart, and lung, was recorded.

### α-NETA repeat dosing toxicity

Mice on the C57/BL6 background received daily treatment of α-NETA (formulated in 10% captisol) at the following doses: 300, 100, 30, 0 mg/kg by oral gavage (200 ul/dose, 3 mice/group) for 14 days. Mouse survival was monitored and body weight was measured daily for the length of the study. On day 14 the mice were euthanized and the wet weight for the following tissues: kidney, brain, spleen, liver, heart, and lung, was recorded.

### Ames test

Ames test was completed using the AMES-MOD ISO 96 well format assay, Version 1.1 (Environmental Bio-Detection Products Inc.) according to manufacturer’s instructions. In brief, *Salmonella typhimurium* (TA100) bacterial strain was exposed to the indicated doses of sodium azide (positive control) or α-NETA solubilized in DMSO (1% DMSO/well, 48 wells/dose completed in triplicate). After 2 h incubation the cells were diluted and cultured in media lacking histidine but containing pH indicator to identify revertant cell growth. After 3 days of incubation at 37 °C, the number of wells containing revertant cells was counted per dose.

### Cyp enzyme inhibition

Human liver microsomes were pre-incubated with 7 concentrations (0.1–100 mM, duplicate wells) of α-NETA for 30 min. Cyp-isoform-specific substrates were then added, incubated 10–60 min, substrate concentration quantified by mass spectrometry, and IC50s calculated (Cyprotex/Evotec AG).

### Cyp induction

Pregnane X receptor (PXR) activation was used as a marker of Cyp induction. An engineered human hepatoma cell line with a PXR luciferase reporter was incubated with the indicated concentrations of α-NETA, Rifampicin (positive control), or DMSO (negative control), and luciferase activity assessed (Cyprotex/Evotec AG).

### hERG inhibition

A patch-clamp assay with single CHO-hERG cell transfectants were used to quantify potential α-NETA-dependent hERG inhibition (2–3 cells per compound concentration). Basal hERG current was measured, and then α-NETA (0.008, 0.04, 0.2, 1, 5, 25 uM), Quinidine (positive control, a known hERG inhibitor) or DMSO (negative control) was added. The cell was depolarized, and the hERG tail current was measured, and IC50 determined (Cyprotex/Evotec AG).

### Statistical methods

All data are presented as mean with error calculated as SEM. Statistical comparisons were made using GraphPad Prism Software (San Diego, CA, USA). Data was analyzed as indicated by a two tailed student’s t-test when comparing two groups or a one-way analysis of variance (ANOVA) with either post-hoc Dunnett’s multiple comparison test or Kruskal Wallis test for multiple group comparisons. An F-test (extra sum of squares) was used to compare the geometric features of the 4^th^ order polynomial curves fit to the clinical EAE data. Fisher’s exact test was used to analyze the 2 × 2 contingency table (Table 4). A p value of <0.05 was considered statistically significant.

### Synthesis of α-NETA analogues

#### General methods

TLCs were run on pre-coated Silica Gel 60F_254_ plates from MilliporeSigma (Burlington, MA, USA) and observed under UV light. Column chromatography was done using a CombiFlash Rf + Lumen chromatography system from Teledyne ISCO (Lincon, NE, USA). For verification of the product and purity analysis, the LC-MS was taken on an Agilent 6490 iFunnel Triple Quadrupole Mass Spectrometer from Agilent Technologies Inc. (Santa Clara, CA, USA). The ^1^H (400 MHz) and^13^C (101 MHz) NMR spectra were taken on an Agilent 400-MR NMR spectrometer from Agilent Technologies Inc. (Santa Clara, CA, USA). Chemical shifts (δ) are expressed in ppm, coupling constants (J) are expressed in Hertz, and splitting patterns are described as follows: s = singlet; d = doublet; t = triplet; q = quartet; br = broad; m = multiplet; dd = doublet of doublets; dt = doublet of triplets; td = triplet of doublets; ddd = doublet of doublet of doublets. All reagents and solvents were purchased from either Sigma-Aldrich (St. Louis, MO, USA) or Fisher Scientific (Hampton, NH, USA) and used without further purification. Additional information can be found in the associated online Supplementary Information.

#### General synthesis of tertiary amines 3–20

In a two-neck round-bottom flask, fitted with a reflux condenser was suspended aryl methyl ketone/cyclohexyl methyl ketone **1** (Table 2, 1.0 eq, 47 mmol) in ethanol (15 mL). Then disubstituted amine HCl salts **2** (1.4 eq, 65 mmol) and paraformaldehyde (1.4 eq, 65 mmol) were added. The mixture was stirred at room temperature for 5–10 min and conc. HCl (~0.6 mL) was added. The resulting mixture was stirred at refluxed for 18–24 h. The progress of the reaction was monitored by TLC. After cooling the mixture to ambient temperature, acetone (~50 mL) was added with continues stirring. The mixture was further cooled to −20 °C and the resulting solid was collected by filtration, washed with child acetone and dried in a vacuum for 1 h. The wet solid was dissolved in distilled water (~100 mL), and pH was adjusted to 9–11 by adding a saturated solution of Na_2_CO_3_. After stirring the solution at room temperature for 30 min, extracted with ethyl acetate (2 × 30 mL). combined organic layer was washed with water, brine, dried over Na_2_SO_4_, filtered and evaporated to dryness to afford a yellow oil, that was further purified on silica gel column with 0–5% methanol in dichloromethane that contains 1% triethyl amine to afford the desired compound as colorless to pale yellow oil.

For compounds 4, 5, 10, 11 and 18, (Table 2) similar procedure was followed except the reaction was carried out in seal tube at 100 °C for 24 h.

#### General synthesis of quaternary ammonium salts 21–33

The tertiary amine **21** (Table 3, 1.0 eq, 1.0 mmol) was suspended in ethanol (5 mL) in a 14 mL glass vial then methyl iodide (1.2 eq, 1.2 mmol) was added dropwise at ambient temperature and stirred for 24 h, during which time the a white solid precipitated. The solid was collected by filtration and washed with copious amounts of ethanol followed by diethyl ether. The compounds were further purified by stirring them in mixture of acetonitrile and diethyl ether for overnight. The desired compounds were obtained as off-white to pale brown solids.

## Supplementary information

Supplementary Information

## Data Availability

The datasets generated during and/or analysed during the current study are available from the corresponding author on reasonable request.
