# Supplementary material for: Novel CMKLR1 Inhibitors for Application in Demyelinating Disease
Source: Sci Rep. 2019 May 9;9:7178. doi: 10.1038/s41598-019-43428-8 (PMC6509344; doi:10.1038/s41598-019-43428-8)
Supplement: Supplementary file 1 — Supplementary Information [file 41598_2019_43428_MOESM1_ESM.pdf]

Supplementary Information

Novel CMKLR1 Inhibitors for Application in Demyelinating Disease

Vineet Kumar[1,4], Melissa LaJevic[3,4], Mallesh Pandrala[1], Sam A. Jacobo[1], Sanjay V. Malhotra[1,2*], and Brian A. Zabel[3*]

[1]Department of Radiation Oncology and [2]Radiology, Stanford University, Palo Alto, California, USA. [3]Palo Alto Veterans Institute for Research, Veterans Affairs Palo Alto Health Care System, Palo Alto, California, USA. [4]These authors contributed equally to this work. [*]e-mail: svmalhotra@stanford.edu or bazabel@stanford.edu

1. Chemistry experimental

General methods: TLCs were run on pre-coated Silica Gel $60F_{254}$ plates from MilliporeSigma (Burlington, MA, USA) and observed under UV light. Column chromatography was done using a CombiFlash Rf+ Lumen chromatography system from Teledyne ISCO (Lincon, NE, USA). For verification of the product and purity analysis, the LC–MS was taken on an Agilent 6490 iFunnel Triple Quadrupole Mass Spectrometer from Agilent Technologies Inc. (Santa Clara, CA, USA). The ^{1}H (400 MHz) and^{13}C (101 MHz) NMR spectra were taken on a Agilent 400-MR NMR spectrometer from Agilent Technologies Inc. (Santa Clara, CA, USA). Chemical shifts (δ) are expressed in ppm, coupling constants (J) are expressed in Hertz, and splitting patterns are described as follows: s = singlet; d = doublet; t = triplet; q = quartet; br = broad; m = multiplet; dd = doublet of doublets; dt = doublet of triplets; td = triplet of doublets; ddd = doublet of doublet of doublets. All reagents and solvents were purchased from either Sigma-Aldrich (St. Louis, MO, USA) or Fisher Scientific (Hampton, NH, USA) and used without further purification.

1.1. General synthesis of tertiary amines 3-20:

In a two-neck round-bottom flask, fitted with a reflux condenser was suspended aryl methyl ketone/cyclohexyl methyl ketone **1** (Table **2**, 1.0 eq, 47 mmol) in ethanol (15 mL). Then disubstituted amine HCl salts **2** (1.4 eq, 65 mmol) and paraformaldehyde (1.4 eq, 65 mmol) were added. The mixture was stirred at room temperature for 5-10 min and conc. HCl (~0.6 mL) was added. The resulting mixture was stirred at refluxed for 18-24 h. The progress of the reaction was monitored by TLC. After cooling the mixture to ambient temperature, acetone (~ 50 mL) was added with continues stirring. The mixture was further cooled to -20 °C and the resulting solid was collected by filtration, washed with child acetone and dried in a vacuum for 1h. The wet solid was dissolved in distilled water (~100 mL), and pH was adjusted to 9-11 by adding a saturated solution of Na_2CO_3. After stirring the solution at room temperature for 30 min, extracted with ethyl acetate (2 X 30 mL). combined organic layer was washed with water, brine, dried over Na_2SO_4, filtered and evaporated to dryness to afford a yellow oil, that was further purified on silica gel column with 0-5% methanol in dichloromethane that contains 1% triethyl amine to afford the desired compound as colorless to pale yellow oil.

For compounds 4, 5, 10, 11 and 18, (Table **2**) similar procedure was followed except the reaction was carried out in seal tube at 100 °C for 24h.

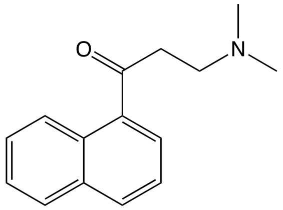

3: Obtained in 51% yield as pale yellow oil. **^{1}H NMR** (400 MHz, Chloroform-*d*) δ 8.60 – 8.53 (m, 1H), 8.01 – 7.95 (m, 1H), 7.90 – 7.83 (m, 2H), 7.61 – 7.44 (m, 3H), 3.23 (dd, *J* = 7.8, 6.8 Hz, 2H), 2.81 (dd, *J* = 7.7, 6.9 Hz, 2H), 2.28 (s, 6H). **^{13}C NMR** (101 MHz, cdcl₃) δ 203.48, 136.11, 133.93, 132.48, 130.09, 128.34, 127.80, 127.26, 126.41, 125.79, 124.33, 54.71, 45.47, 40.54. LC-MS (ESI-TOF): m/z 228.30 ([$C_{15}H_{17}NO$ + H]$^+$ calcd 228.13).

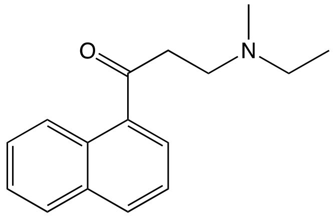

4: Obtained in 44% yield as colorless oil. **^{1}H NMR** (400 MHz, Chloroform-*d*) δ 8.57 (ddt, *J* = 8.6, 1.5, 0.8 Hz, 1H), 7.96 (dt, *J* = 8.3, 1.1 Hz, 1H), 7.86 (dt, *J* = 7.5, 1.4 Hz, 2H), 7.62 – 7.42 (m, 3H), 3.23 (dd, *J* = 7.8, 6.8 Hz, 2H), 2.88 (dd, *J* = 7.8, 6.8 Hz, 2H), 2.45 (q, *J* = 7.2 Hz, 2H), 2.26 (s, 3H), 1.04 (t, *J* = 7.2 Hz, 3H). ^{13}C NMR (101 MHz, cdcl₃) δ 203.79, 136.19, 133.94, 132.44, 130.12, 128.34, 127.77, 127.26, 126.40, 125.85, 124.33, 52.46, 51.39, 41.56, 40.21, 12.33. LC-MS (ESI-TOF): m/z 242.30 ([$C_{16}H_{19}NO$ + H]$^+$ calcd 242.16).

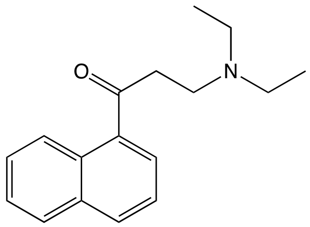

5: Obtained in 14% yield as light brown oil. **^{1}H NMR** (400 MHz, Chloroform-*d*) δ 8.62 – 8.51 (m, 1H), 8.02 – 7.92 (m, 1H), 7.86 (ddt, *J* = 8.0, 2.1, 1.0 Hz, 2H), 7.63 – 7.41 (m, 4H), 3.28 – 3.15 (m, 2H), 3.03 – 2.91 (m, 2H), 2.55 (q, *J* = 7.2 Hz, 4H), 1.01 (t, *J* = 7.2 Hz, 6H). ^{13}C NMR (101 MHz, cdcl₃) δ 204.13, 136.26, 133.94, 132.41, 130.14, 128.33, 127.73, 127.29, 126.38, 125.89, 124.33, 48.35, 46.87, 39.95, 11.74. LC-MS (ESI-TOF): m/z 256.30 ([$C_{17}H_{21}NO$ + H]$^+$ calcd 256.16).

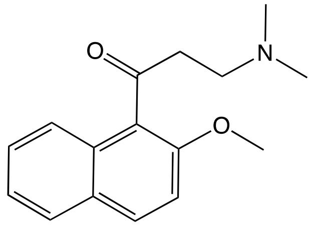

6: Obtained in 58% yield as pale yellow oil. **^{1}H NMR** (400 MHz, Chloroform-*d*) δ 7.87 (dt, *J* = 9.1, 0.6 Hz, 1H), 7.78 (ddt, *J* = 8.2, 1.3, 0.6 Hz, 1H), 7.72 (dq, *J* = 8.5, 0.9 Hz, 1H), 7.45 (ddd, *J* = 8.4, 6.8, 1.4 Hz, 1H), 7.35 (ddd, *J* = 8.1, 6.8, 1.2 Hz, 1H), 7.29 – 7.23 (m, 1H), 3.95 (s, 3H), 3.18 – 3.06 (m, 2H), 2.78 (dd, *J* = 7.9, 6.9 Hz, 2H), 2.25 (s, 6H). **^{13}C NMR** (101 MHz, cdcl₃) δ 206.38, 153.75, 131.37, 130.59, 128.82, 128.07, 127.56, 124.91, 124.07, 123.72, 112.65, 56.34, 53.99, 45.39, 43.35. LC-MS (ESI-TOF): m/z 258.20 ([$C_{16}H_{19}NO_2$ + H]$^+$ calcd 258.14).

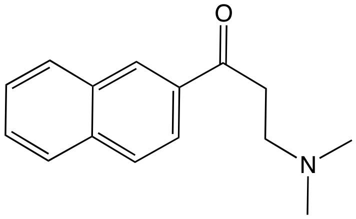

7: Obtained in 38% yield as off white semi solid. **^{1}H NMR** (400 MHz, Chloroform-*d*) δ 8.50 – 8.39 (m, 1H), 8.02 (dd, *J* = 8.6, 1.8 Hz, 1H), 7.94 (ddd, *J* = 7.9, 1.5, 0.7 Hz, 1H), 7.90 – 7.79 (m, 2H), 7.62 – 7.45 (m, 2H), 3.26 (dd, *J* = 7.9, 6.8 Hz, 2H), 2.80 (dd, *J* = 7.8, 6.9 Hz, 2H), 2.30 (s, 6H). **^{13}C NMR** (101 MHz, $cdcl_3$) δ 199.04, 135.54, 134.22, 132.48, 129.69, 129.56, 128.44, 128.43, 127.74, 126.74, 123.83, 54.55, 45.59, 37.02. LC-MS (ESI-TOF): m/z 228.30 ($[C_{15}H_{17}NO + H]^+$ calcd 228.13).

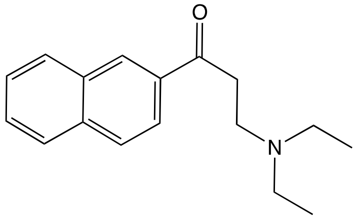

8: Obtained in 44% yield as brown oil. **^{1}H NMR** (400 MHz, Chloroform-*d*) δ 8.52 – 8.41 (m, 1H), 8.02 (dd, *J* = 8.6, 1.8 Hz, 1H), 7.94 (ddd, *J* = 7.9, 1.5, 0.7 Hz, 1H), 7.90 – 7.79 (m, 2H), 7.62 – 7.47 (m, 2H), 3.33 – 3.17 (m, 2H), 3.04 – 2.87 (m, 2H), 2.59 (q, *J* = 7.1 Hz, 4H), 1.06 (t, *J* = 7.1 Hz, 6H). **^{13}C NMR** (101 MHz, $cdcl_3$) δ 199.69, 135.53, 134.35, 132.50, 129.73, 129.54, 128.42, 128.39, 127.74, 126.73, 123.84, 48.04, 47.04, 36.54, 11.87. LC-MS (ESI-TOF): m/z 256.30 ($[C_{17}H_{21}NO + H]^+$ calcd 256.16).

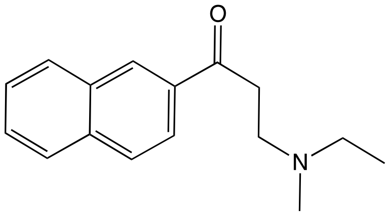

9: Obtained in 62% yield as colorless oil. **^{1}H NMR** (400 MHz, Chloroform-*d*) δ 8.50 – 8.43 (m, 1H), 8.02 (dd, *J* = 8.6, 1.8 Hz, 1H), 7.94 (ddt, *J* = 7.9, 1.4, 0.7 Hz, 1H), 7.90 – 7.80 (m, 2H), 7.56 (dddd, *J* = 18.8, 8.2, 6.9, 1.4 Hz, 2H), 3.34 – 3.21 (m, 2H), 2.89 (dd, *J* = 8.1, 6.8 Hz, 2H), 2.50 (q, *J* = 7.2 Hz, 2H), 2.30 (s, 3H), 1.09 (t, *J* = 7.2 Hz, 3H). **^{13}C NMR** (101 MHz, $cdcl_3$) δ 199.32, 135.55, 134.29, 132.51, 129.70, 129.55, 128.43, 128.41, 127.74, 126.73, 123.83, 52.19, 51.48, 41.72, 36.75, 12.37. LC-MS (ESI-TOF): m/z 242.20 ($[C_{16}H_{19}NO + H]^+$ calcd 242.15).

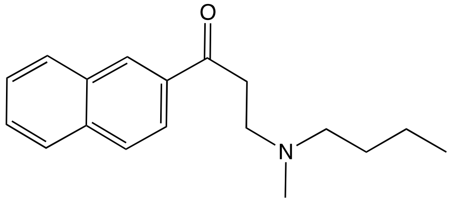

10: Obtained in 63% yield as colorless oil. **^{1}H NMR** (400 MHz, Chloroform-*d*) δ 8.48 (d, *J* = 1.3 Hz, 1H), 8.03 (dd, *J* = 8.6, 1.8 Hz, 1H), 7.96 (ddt, *J* = 7.9, 1.5, 0.7 Hz, 1H), 7.92 – 7.81 (m, 2H), 7.57 (dddd, *J* = 18.7, 8.2, 6.9, 1.4 Hz, 2H), 3.35 – 3.20 (m, 2H), 2.89 (dd, *J* = 8.1, 6.8 Hz, 2H), 2.49 – 2.36 (m, 2H), 2.31 (s, 3H), 1.55 – 1.40 (m, 2H),

1.40 – 1.25 (m, 2H), 0.91 (t, *J* = 7.3 Hz, 3H). **^{13}C NMR** (101 MHz, cdcl$_3$) δ 199.46, 135.54, 134.29, 132.50, 129.72, 129.56, 128.44, 128.42, 127.75, 126.74, 123.84, 57.64, 52.65, 42.37, 36.71, 29.52, 20.70, 14.10. LC-MS (ESI-TOF): m/z 270.30 ([$C_{18}H_{23}NO$ + H]$^+$ calcd 270.18).

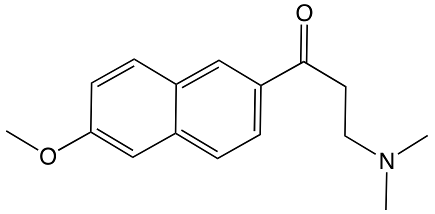

11: Obtained in 65% yield as off white solid. **^{1}H NMR** (400 MHz, Chloroform-*d*) δ 8.41 (d, *J* = 1.7 Hz, 1H), 8.00 (ddd, *J* = 8.6, 1.9, 0.6 Hz, 1H), 7.85 (dt, *J* = 8.9, 0.6 Hz, 1H), 7.76 (dt, *J* = 8.6, 0.6 Hz, 1H), 7.29 – 7.11 (m, 2H), 3.94 (d, *J* = 0.6 Hz, 3H), 3.33 – 3.20 (m, 2H), 2.82 (t, *J* = 7.4 Hz, 2H), 2.33 (d, *J* = 0.6 Hz, 6H). **^{13}C NMR** (101 MHz, cdcl$_3$) δ 198.71, 159.74, 137.27, 132.31, 131.13, 129.59, 127.81, 127.13, 124.58, 119.71, 105.71, 55.41, 54.61, 45.52, 36.78. LC-MS (ESI-TOF): m/z 258.30 ([$C_{16}H_{19}NO_2$ + H]$^+$ calcd 258.14)

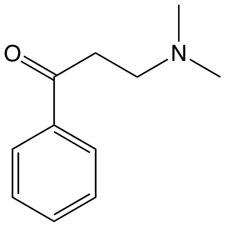

12: Obtained in 67.5% yield as colorless oil. **^{1}H NMR** (400 MHz, Chloroform-*d*) δ 7.98 – 7.92 (m, 2H), 7.58 – 7.51 (m, 1H), 7.48 – 7.40 (m, 2H), 3.21 – 3.08 (m, 2H), 2.75 (dd, *J* = 7.9, 6.8 Hz, 2H), 2.28 (s, 6H). **^{13}C NMR** (101 MHz, cdcl$_3$) δ 199.11, 133.06, 128.59, 128.02, 54.35, 45.53, 36.91. LC-MS (ESI-TOF): m/z 178.30 ([$C_{11}H_{15}NO$ + H]$^+$ calcd 178.12).

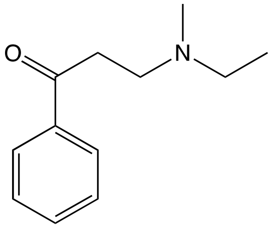

13: Obtained in 63% yield as light brown oil. **^{1}H NMR** (400 MHz, Chloroform-*d*) δ 7.96 – 7.86 (m, 2H), 7.55 – 7.46 (m, 1H), 7.45 – 7.35 (m, 2H), 3.11 (dd, *J* = 8.1, 6.7 Hz, 2H), 2.79 (dd, *J* = 8.1, 6.7 Hz, 2H), 2.43 (q, *J* = 7.2 Hz, 2H), 2.23 (s, 3H), 1.02 (t, *J* = 7.2 Hz, 3H). **^{13}C NMR** (101 MHz, cdcl$_3$) δ 199.30, 136.92, 132.98, 128.56, 128.54, 127.98, 127.97, 51.97, 51.41, 41.66, 36.59, 12.34. LC-MS (ESI-TOF): m/z 192.30 ([$C_{12}H_{17}NO$ + H]$^+$ calcd 192.13).

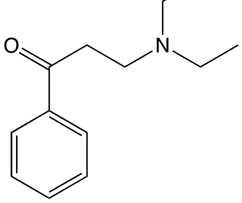

14: Obtained in 29% yield as colorless oil. **^{1}H NMR** (400 MHz, Chloroform-*d*) δ 8.01 – 7.91 (m, 2H), 7.60 – 7.51 (m, 1H), 7.50 – 7.41 (m, 2H), 3.18 – 3.08 (m, 2H), 2.97 – 2.89 (m, 2H), 2.58 (q, *J* = 7.1 Hz, 4H), 1.05 (t, *J* = 7.1 Hz, 6H). ^{13}C NMR (101 MHz, cdcl$_3$) δ 199.74, 137.05, 132.96, 128.55, 128.02, 47.87, 47.02, 36.43, 11.84. LC-MS (ESI-TOF): m/z 206.30 ([$C_{13}H_{19}NO$ + H]$^+$ calcd 206.15).

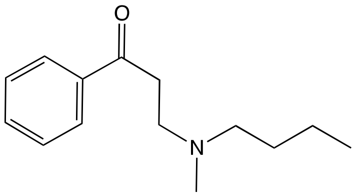

15: Obtained in 73% yield as colorless oil. **^{1}H NMR** (400 MHz, Chloroform-*d*) δ 7.98 – 7.90 (m, 2H), 7.57 – 7.49 (m, 1H), 7.48 – 7.39 (m, 2H), 3.18 – 3.08 (m, 2H), 2.85 – 2.76 (m, 2H), 2.41 – 2.32 (m, 2H), 2.25 (s, 3H), 1.50 – 1.38 (m, 2H), 1.34 – 1.23 (m, 2H), 0.89 (t, *J* = 7.3 Hz, 3H). **^{13}C NMR** (101 MHz, cdcl3) δ 199.45, 137.00, 132.96, 128.55, 128.01, 57.56, 52.50, 42.29, 36.60, 29.49, 20.63. LC-MS (ESI-TOF): m/z 220.30 ([$C_{14}H_{21}NO$ + H]$^+$ calcd 220.16).

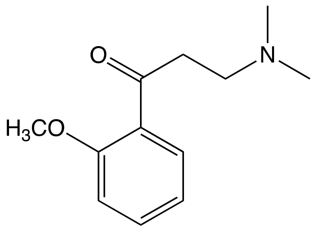

16: Obtained in 44% yield as colorless oil. **^{1}H NMR** (400 MHz, Chloroform-*d*) δ 7.66 (ddd, *J* = 7.7, 1.8, 0.4 Hz, 1H), 7.43 (ddd, *J* = 8.3, 7.3, 1.8 Hz, 1H), 7.01 – 6.90 (m, 2H), 3.88 (d, *J* = 0.3 Hz, 3H), 3.21 – 3.09 (m, 2H), 2.72 – 2.63 (m, 2H), 2.25 (d, *J* = 0.5 Hz, 6H). **^{13}C NMR** (101 MHz, cdcl3) δ 201.44, 158.44, 133.34, 130.29, 128.36, 120.62, 111.43, 55.44, 54.46, 45.47, 42.06. LC-MS (ESI-TOF): m/z 208.30 ([$C_{12}H_{17}NO_2$ + H]$^+$ calcd 208.13).

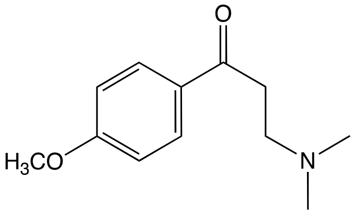

17 : Obtained in 51% yield as colorless oil. **^{1}H NMR** (400 MHz, Chloroform-d) δ 7.92 (dd, J = 9.0, 0.9 Hz, 2H), 6.90 (dd, J = 9.0, 0.9 Hz, 2H), 3.83 (d, J = 1.0 Hz, 3H), 3.13 – 3.02 (m, 2H), 2.71 (td, J = 7.4, 0.8 Hz, 2H), 2.26 (d, J = 0.9 Hz, 6H). **^{13}C NMR** (101 MHz, cdcl3) δ 197.63, 163.42, 130.28, 130.02, 113.70, 55.42, 54.58, 45.50, 36.56. LC-MS (ESI-TOF): m/z 208.30 ([$C_{12}H_{17}NO_2$ + H]$^+$ calcd 208.13).

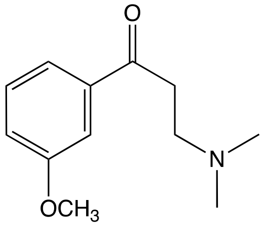

18: Obtained in 53% yield as pale yelow oil. **^{1}H NMR** (400 MHz, Chloroform-*d*) δ 7.52 (dddd, *J* = 7.6, 1.5, 1.0, 0.4 Hz, 1H), 7.46 (dd, *J* = 2.7, 1.6 Hz, 1H), 7.34 (dd, *J* = 8.2, 7.7 Hz, 1H), 7.08 (dddd, *J* = 8.2, 2.6, 1.0, 0.5 Hz, 1H), 3.82 (dd, *J* = 1.4, 0.5 Hz, 4H), 3.11 (dd, *J* = 7.7, 6.9 Hz, 2H), 2.72 (dd, *J* = 7.9, 6.8 Hz, 2H), 2.26 (s, 6H). ^{13}C NMR (101 MHz, cdcl3) δ 198.89, 159.80, 138.28, 129.55, 120.64, 119.50, 112.26, 55.38, 54.41, 45.49, 37.01, 26.68. LC-MS (ESI-TOF): m/z 208.20 ([$C_{12}H_{17}NO_2$ + H]$^+$ calcd 208.13).

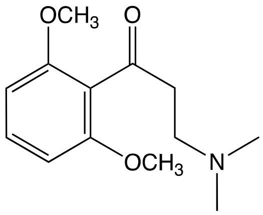

19: Obtained in 54% yield as colorless oil. **^{1}H NMR** (400 MHz, Chloroform-*d*) δ 7.28 – 7.18 (m, 1H), 6.52 (dd, *J* = 8.4, 0.7 Hz, 2H), 3.75 (d, *J* = 0.9 Hz, 6H), 2.92 (ddd, *J* = 8.3, 6.7, 0.6 Hz, 2H), 2.74 – 2.63 (m, 2H), 2.22 (d, *J* = 0.8 Hz, 6H). ^{13}C NMR (101 MHz, cdcl$_3$) δ 203.66, 156.68, 130.59, 120.27, 103.92, 103.86, 55.77, 53.38, 45.33, 42.96. LC-MS (ESI-TOF): m/z 238.30 ([$C_{13}H_{19}NO_3$ + H]$^+$ calcd 238.14).

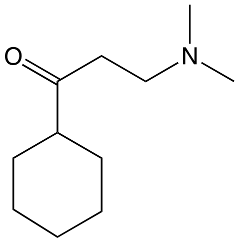

20: Obtained in 28% yield as pale yellow oil. **^{1}H NMR** (400 MHz, Chloroform-*d*) δ 2.61 – 2.54 (m, 2H), 2.54 – 2.48 (m, 2H), 2.37 – 2.25 (m, 1H), 2.18 (s, 6H), 1.88 – 1.68 (m, 4H), 1.67 – 1.57 (m, 1H), 1.37 – 1.07 (m, 5H).**^{13}C NMR** (101 MHz, cdcl$_3$) δ 212.86, 53.83, 50.99, 45.43, 38.86, 28.39, 25.81, 25.62. LC-MS (ESI-TOF): m/z 184.30 ([$C_{11}H_{21}NO$ + H]$^+$ calcd 184.16).

1.2. General synthesis of quaternary ammonium salts 22-33:

The tertiary amine **21** (Table **3**, 1.0 eq, 1.0 mmol) was suspended in ethanol (5 mL) in a 14 mL glass vial then methyl iodide (1.2 eq, 1.2 mmol) was added dropwise at ambient temperature and stirred for 24h, during which time the a white solid precipitated. The solid was collected by filtration and washed with copious amounts of ethanol followed by diethyl ether. The compounds were further purified by stirring them in mixture of acetonitrile and diethyl ether for overnight. The desired compounds were obtained as off-white to pale brown solids.

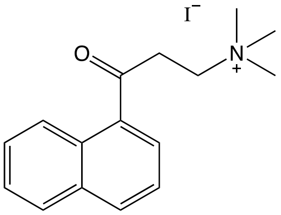

22: Obtained in 58% yield as off white solid. **^{1}H NMR** (400 MHz, DMSO-*d*$_6$) δ 8.62 – 8.54 (m, 1H), 8.32 (dd, *J* = 7.3, 1.2 Hz, 1H), 8.20 (dt, *J* = 8.4, 1.0 Hz, 1H), 8.06 – 7.99 (m, 1H), 7.71 – 7.53 (m, 3H), 3.79 (s, 4H), 3.16 (s, 9H). **^{13}C NMR** (101 MHz, dmso) δ 200.22, 134.27, 133.93, 133.83, 129.84, 129.08, 128.56, 126.97, 125.68, 125.20, 61.47 (t, *J* = 4 Hz), 54.86 (t, *J* = 4 Hz), 53.11 (t, *J* = 4 Hz), 35.39. LC-MS (ESI-TOF): m/z 242.30 ($C_{16}H_{20}NO^+$ calcd 242.15).

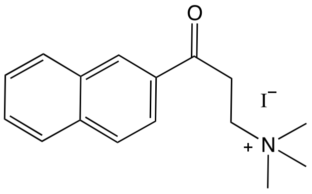

23: Obtained in 34% yield as off white solid. **^{1}H NMR** (400 MHz, DMSO-d_6) δ 8.81 (q, *J* = 1.2 Hz, 1H), 8.19 – 8.11 (m, 1H), 8.11 – 8.00 (m, 3H), 7.76 – 7.61 (m, 2H), 3.93 – 3.81 (m, 2H), 3.77 (ddd, *J* = 8.3, 6.9, 1.9 Hz, 2H), 3.18 (s, 9H). **^{13}C NMR** (101 MHz, dmso) δ 196.61, 135.71, 133.68, 132.51, 130.69, 129.92, 129.36, 128.80, 128.23, 127.63, 123.88, 61.38, 53.07, 32.71. LC-MS (ESI-TOF): m/z 242.20 ($C_{16}H_{20}NO^+$ calcd 242.15).

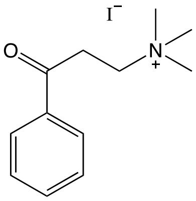

24: Obtained in 76% yield as white solid. **^{1}H NMR** (400 MHz, DMSO-d_6) δ 8.11 – 8.04 (m, 2H), 7.74 – 7.67 (m, 1H), 7.62 – 7.54 (m, 2H), 3.71 (s, 4H), 3.15 (s, 9H). **^{13}C NMR** (101 MHz, dmso) δ 196.69, 136.35, 134.21, 129.18, 128.57, 61.28, 53.06, 53.03, 52.99, 32.65. LC-MS (ESI-TOF): m/z 192.30 ($C_{12}H_{18}NO^+$ calcd 192.14).

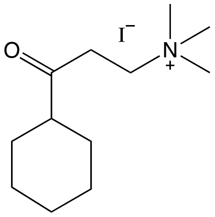

25: Obtained in 30% yield as yellowish solid. **^{1}H NMR** (400 MHz, DMSO-d_6) δ 3.49 (dd, *J* = 8.3, 6.9 Hz, 2H), 3.16 – 3.07 (m, 4H), 3.04 (s, 9H), 2.43 (dd, *J* = 7.1, 3.6 Hz, 1H), 1.91 – 1.67 (m, 4H), 1.68 – 1.57 (m, 1H), 1.32 – 1.10 (m, 5H). **^{13}C NMR** (101 MHz, dmso) δ 210.13, 60.86 (t, *J* = 3Hz), 54.83 (t, *J* = 4Hz), 52.84(t, *J* = 4Hz), 50.22, 33.92, 28.18, 25.82, 25.46. LC-MS (ESI-TOF): m/z 198.00 ($C_{12}H_{24}NO^+$ calcd 198.19).

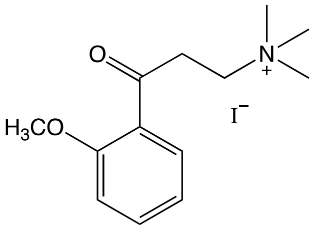

26: Obtained in 50% yield as Yellowish solid. **^{1}H NMR** (400 MHz, DMSO-d_6) δ 7.66 (dd, *J* = 7.7, 1.8 Hz, 1H), 7.60 (ddd, *J* = 8.4, 7.3, 1.8 Hz, 1H), 7.23 (dd, *J* = 8.5, 0.9 Hz, 1H), 7.07 (td, *J* = 7.5, 1.0 Hz, 1H), 3.93 (s, 3H), 3.68 (dd, *J* = 8.3, 6.6 Hz, 2H), 3.51 (t, *J* = 7.2 Hz, 2H), 3.11 (s, 13H). **^{13}C NMR** (101 MHz, dmso) δ 197.77, 158.94, 134.92, 130.15, 127.10, 120.93, 113.07, 61.23 (t, *J* = 3 Hz), 56.42, 54.85 (t, *J* = 4 Hz), 52.98 (t, *J* = 4 Hz), 37.09. LC-MS (ESI-TOF): m/z 222.30 ($C_{13}H_{20}NO_2^+$ calcd 222.15).

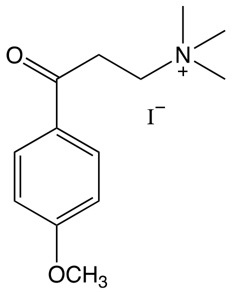

27: Obtained in 64% yield as off white solid. **^{1}H NMR** (400 MHz, DMSO-*d*$_6$) δ 8.05 (d, *J* = 8.9 Hz, 2H), 7.09 (d, *J* = 8.9 Hz, 2H), 3.87 (s, 3H), 3.76 – 3.58 (m, 4H), 3.14 (s, 7H), 3.12 – 3.09 (m, 2H). **^{13}C NMR** (101 MHz, dmso) δ 195.00, 164.00, 130.96, 130.94, 129.34, 114.37, 114.32, 61.45, 56.13, 54.85 (t, *J* = 4 Hz), 53.02 (t, *J* = 4 Hz), 32.20. LC-MS (ESI-TOF): m/z 222.30 ($C_{13}H_{20}NO_2^+$ calcd 222.15).

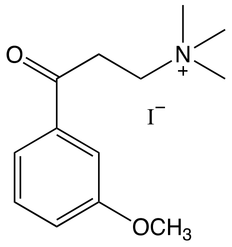

28: Obtained in 56% yield as off white solid. **^{1}H NMR** (400 MHz, DMSO-*d*$_6$) δ 7.67 (ddd, *J* = 7.7, 1.6, 1.0 Hz, 1H), 7.56 (dd, *J* = 2.7, 1.5 Hz, 1H), 7.50 (dd, *J* = 8.2, 7.7 Hz, 1H), 7.28 (ddd, *J* = 8.2, 2.7, 0.9 Hz, 1H), 3.85 (s, 3H), 3.69 (s, 4H), 3.14 (s, 7H), 3.11 (s, 3H). **^{13}C NMR** (101 MHz, dmso) δ 196.51, 159.89, 137.74, 130.38, 121.01, 119.91, 113.50, 61.32, 55.97, 54.85 (t, *J* = 4 Hz), 53.04 (t, *J* = 4 Hz), 32.79. LC-MS (ESI-TOF): m/z 222.30 ($C_{13}H_{20}NO_2^+$ calcd 222.15).

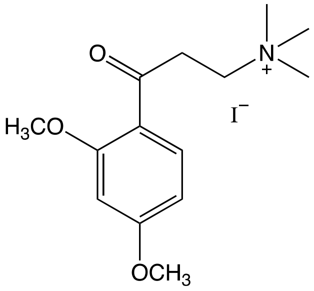

29: Obtained in 74% yield as baby pink solid. **^{1}H NMR** (400 MHz, DMSO-*d*6) δ 7.74 (d, *J* = 8.7 Hz, 1H), 6.70 (d, *J* = 2.3 Hz, 1H), 6.68 – 6.60 (m, 1H), 3.95 (s, 3H), 3.86 (s, 3H), 3.65 (dd, *J* = 8.5, 6.8 Hz, 2H), 3.44 (t, *J* = 7.5 Hz, 2H), 3.11 (d, *J* = 0.9 Hz, 11H). **^{13}C NMR** (101 MHz, dmso) δ 195.02, 165.26, 161.52, 132.55, 119.54, 106.63, 98.92, 61.49, 56.50, 56.21, 54.81 (t, *J* = 4 Hz), 52.95 (t, *J* = 4 Hz), 36.88. LC-MS (ESI-TOF): m/z 252.30 ($C_{14}H_{22}NO_3^+$ calcd 252.16).

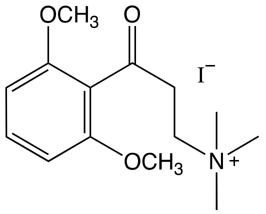

30: Obtained in 61% yield as off white solid. **^{1}H NMR** (400 MHz, DMSO-*d*$_6$) δ 7.40 (t, *J* = 8.4 Hz, 1H), 6.76 (d, *J* = 8.4 Hz, 2H), 3.78 (s, 6H), 3.68 (t, *J* = 7.5 Hz, 2H), 3.27 (t, *J* = 7.4 Hz, 2H), 3.09 (s, 9H). **^{13}C NMR** (101 MHz, dmso) δ 200.28, 156.68, 132.05, 119.06, 104.91, 60.29, 56.46, 54.85 (t, *J* = 4 Hz), 52.80, 38.03. LC-MS (ESI-TOF): m/z 252.20 ($C_{14}H_{22}NO_3^+$ calcd 252.16).

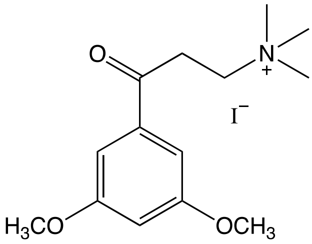

31: Obtained in 58% yield as off white solid. **^{1}H NMR** (400 MHz, DMSO-*d*6) δ 7.19 (d, *J* = 2.3 Hz, 2H), 6.83 (t, *J* = 2.3 Hz, 1H), 3.83 (s, 6H), 3.67 (d, *J* = 1.1 Hz, 4H), 3.15 (s, 9H). **^{13}C NMR** (101 MHz, dmso) δ 196.37, 161.07, 138.30, 106.50, 105.73, 61.26, 56.14, 54.82 (t, *J* = 4 Hz), 53.02, 32.86. LC-MS (ESI-TOF): m/z 252.30 ($C_{14}H_{22}NO_3^+$ calcd 252.16).

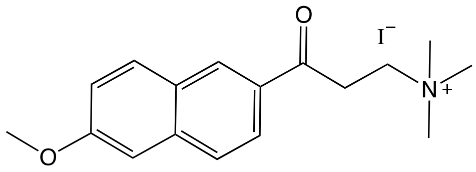

32: Obtained in 33% yield as off white solid. **^{1}H NMR** (400 MHz, DMSO-*d*6) δ 8.75 (d, *J* = 1.7 Hz, 1H), 8.10 – 7.99 (m, 2H), 7.95 (d, *J* = 8.7 Hz, 1H), 7.45 (d, *J* = 2.6 Hz, 1H), 7.31 (dd, *J* = 9.0, 2.5 Hz, 1H), 3.93 (s, 3H), 3.87 – 3.67 (m, 4H), 3.19 (s, 9H). **^{13}C NMR** (101 MHz, dmso) δ 196.19, 160.01, 137.63, 131.63, 131.60, 130.64, 127.77, 127.53, 124.56, 120.21, 106.62, 61.48, 55.95, 54.86 (t, *J* = 3 Hz), 53.09, 32.55. LC-MS (ESI-TOF): m/z 272.20 ($C_{17}H_{22}NO_2^+$ calcd 272.16).

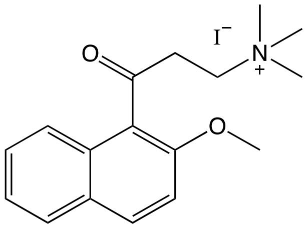

33: Obtained in 57% yield as off white solid. **^{1}H NMR** (400 MHz, DMSO-*d*6) δ 8.17 – 8.07 (m, 1H), 8.01 – 7.91 (m, 1H), 7.63 (dq, *J* = 8.6, 0.9 Hz, 1H), 7.59 (d, *J* = 9.2 Hz, 1H), 7.54 (ddd, *J* = 8.5, 6.8, 1.4 Hz, 1H), 7.43 (ddd, *J* = 8.1, 6.8, 1.2 Hz, 1H), 4.01 (s, 3H), 3.81 (dd, *J* = 8.3, 6.9 Hz, 2H), 3.50 (t, *J* = 7.5 Hz, 2H), 3.12 (d, *J* = 9.7 Hz, 9H). **^{13}C NMR** (101 MHz, dmso) δ 203.04, 154.61, 132.66, 130.03, 128.78, 128.71, 128.25, 124.63, 123.52, 123.36, 114.02, 60.71, 57.12, 54.85(t, J = 4 Hz), 52.90, 38.36. LC-MS (ESI-TOF): m/z 272.30 ($C_{17}H_{22}NO_2^+$ calcd 272.16).

1.3. Synthesis of hydroxyl α-NETA derivatives 34

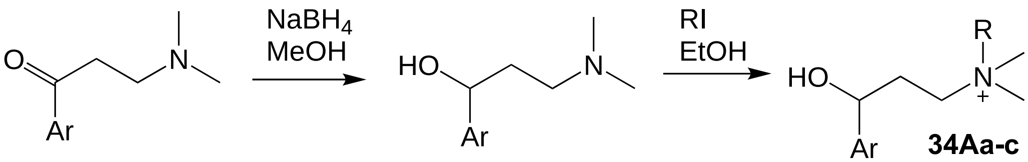

S1A-D

S2A-D

34Aa-c
34Ba-c
34Ca-d
34Da-d

**S1**	**Ar**
A	cylohexyl
B	phenyl
C	a-napthyl
D	b-napthyl

**34 A-D**	**R**
a	Me
b	Et
c	Bu
d	MoE

Scheme S1

Synthesis of S2A-D:

Under a nitrogen atmosphere, the keto amine **S1** (1.0 eq, 8.9 mmol) was taken in methanol (15 mL) in a two-neck round-bottom flask. The mixture was cooled to 0 – 5 °C and then $NaBH_4$ (1.3 eq, 1.15 mmol) was added in small portions over 10 min. The reaction mixture was stirred for 3h at 0 – 5 °C.and the progress of reaction was monitored by TLC. After completion the reaction was quenched with water (2.0 mL). The solvent volume then reduced to 10% on rotary evaporator. The crude obtained was taken into ethyl acetate (150 mL) and then washed with water (50 mL) followed by brine solution (50 mL). The organic layer was dried over Na_2SO_4, filtered and evaporated to dryness to afford the desired compound **S2** as waxy solid.

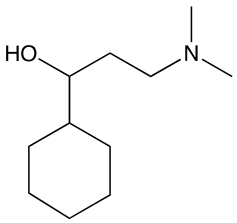

S2A: Obtained in 86% yield as colorless oil. **^{1}H NMR** (400 MHz, Chloroform-*d*) δ 3.49 (ddd, *J* = 9.8, 6.4, 2.5 Hz, 1H), 2.62 (ddd, *J* = 12.4, 11.3, 3.4 Hz, 1H), 2.44 (ddd, *J* = 12.4, 4.3, 3.4 Hz, 1H), 2.24 (s, 6H), 1.98 – 1.87 (m, 1H), 1.80 – 1.61 (m, 4H), 1.61 – 1.43 (m, 2H), 1.37 – 1.07 (m, 4H), 1.07 – 0.91 (m, 2H). **^{13}C NMR** (101 MHz, $cdcl_3$) δ 78.31, 59.40, 45.25, 44.10, 29.11, 28.79, 28.58, 26.64, 26.31, 26.21. LC-MS (ESI-TOF): m/z 186.40 ($[C_{11}H_{23}NO + H]^+$ calcd 186.18).

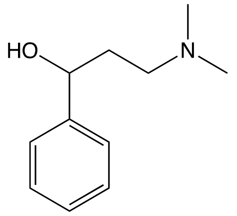

S2B: Obtained in 82% yield as light yellow oil. **^{1}H NMR** (400 MHz, Chloroform-*d*) δ 7.41 – 7.30 (m, 4H), 7.27 – 7.20 (m, 1H), 4.93 (dd, *J* = 7.7, 3.9 Hz, 1H), 2.65 (ddd, *J* = 12.9, 8.9, 4.2 Hz, 1H), 2.51 – 2.43 (m, 1H), 2.30 (s, 6H), 1.89 – 1.74 (m, 2H). **^{13}C NMR** (101 MHz, cdcl$_3$) δ 145.09, 128.14, 126.81, 125.53, 75.75, 58.42, 45.30, 34.51. LC-MS (ESI-TOF): m/z 180.30 ([$C_{11}H_{17}NO$ + H]$^+$ calcd 180.13).

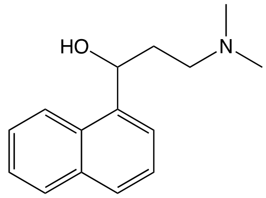

S2C: Obtained in 91% yield as light brown semisolid. **^{1}H NMR** (400 MHz, Chloroform-*d*) δ 8.05 – 7.97 (m, 1H), 7.90 – 7.82 (m, 1H), 7.76 (tt, *J* = 7.9, 0.9 Hz, 2H), 7.55 – 7.39 (m, 3H), 5.71 (dd, *J* = 8.1, 3.1 Hz, 1H), 2.69 (ddd, *J* = 12.7, 9.6, 3.2 Hz, 1H), 2.50 (ddd, *J* = 12.5, 6.4, 3.2 Hz, 1H), 2.34 (s, 6H), 2.14 – 2.01 (m, 1H), 1.98 – 1.85 (m, 1H). **^{13}C NMR** (101 MHz, cdcl$_3$) δ 140.38, 133.73, 130.14, 128.86, 127.31, 125.65, 125.52, 125.17, 122.98, 122.96, 72.56, 58.49, 45.36, 33.28. LC-MS (ESI-TOF): m/z 230.30 ([$C_{15}H_{19}NO$ + H]$^+$ calcd 230.15).

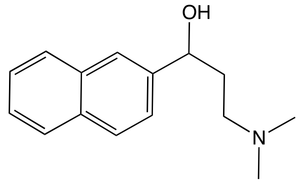

S2D: Obtained in 93% yield as off white solid. **^{1}H NMR** (400 MHz, Chloroform-*d*) δ 7.92 – 7.73 (m, 4H), 7.51 – 7.38 (m, 3H), 5.16 – 5.02 (m, 1H), 2.66 (ddd, *J* = 12.8, 9.2, 3.8 Hz, 1H), 2.52 – 2.43 (m, 1H), 2.30 (s, 6H), 1.98 – 1.79 (m, 2H). **^{13}C NMR** (101 MHz, cdcl$_3$) δ 142.56, 133.39, 132.69, 127.94, 127.81, 127.59, 125.87, 125.41, 124.16, 123.97, 75.80, 58.39, 45.33, 34.41. LC-MS (ESI-TOF): m/z 230.30 ([$C_{15}H_{19}NO$ + H]$^+$ calcd 230.15).

Synthesis of 34A-D:

The tertiary amine **S2** (1.0 eq, 0.8 mmol) was suspended in ethanol (5 mL) in a 14 mL glass vial followed by dropwise addition of alkyl iodide (1.5 eq, 1.26 mmol) at room temperature and the reaction mixture was stirred for 16 – 18h, during which time the a white solid precipitated. The solvent was removed using nitrogen flow to afford semisolid, which was taken in diethyl ether (~25 mL) and stirred for 30 min to obtain free-flow solid. The solid was collected by filtration and washed with copious amounts of diethyl ether and dried in a high vacuum to afford the desired compound as off white solid. For the reaction where butyl iodide was a reactant, similar procedure was followed except the reaction was carried out in seal tube at 65 °C for 24h.

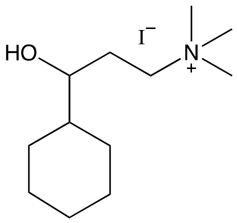

34Aa: Obtained in 60% yield as off white solid. **^{1}H NMR** (400 MHz, DMSO-d_6) δ 4.66 (d, *J* = 5.7 Hz, 1H), 3.43 (td, *J* = 12.4, 5.0 Hz, 1H), 3.37 – 3.26 (m, 2H), 3.21 (dp, *J* = 8.7, 2.8 Hz, 1H), 3.06 (s, 9H), 1.85 – 1.57 (m, 7H), 1.30 – 0.90 (m, 6H). **^{13}C NMR** (101 MHz, dmso) δ 72.12, 64.52, 52.68, 52.64, 52.60, 43.95, 28.99, 28.16, 27.30, 26.56, 26.27, 26.16. LC-MS (ESI-TOF): m/z 200.30 ($C_{12}H_{26}NO^+$ calcd 200.20).

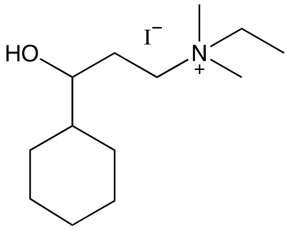

34Ab: Obtained in 87% yield as colorless semisolid. **^{1}H NMR** (400 MHz, DMSO-d_6) δ 4.66 (d, *J* = 5.8 Hz, 1H), 3.40 – 3.32 (m, 2H), 3.32 – 3.13 (m, 3H), 2.98 (s, 6H), 1.89 – 1.67 (m, 4H), 1.67 – 1.52 (m, 3H), 1.35 – 0.87 (m, 9H). **^{13}C NMR** (101 MHz, dmso) δ 72.12, 61.46, 58.74, 50.01, 43.96, 29.00, 28.17, 26.89, 26.56, 26.26, 26.15, 8.23. LC-MS (ESI-TOF): m/z 214.40 ($C_{13}H_{28}NO^+$ calcd 214.22).

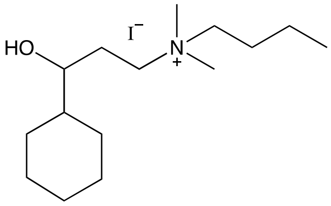

34Ac: Obtained in 81% yield as colorless semisolid. **^{1}H NMR** (400 MHz, DMSO-d_6) δ 4.64 (d, *J* = 5.8 Hz, 1H), 3.44 – 3.16 (m, 5H), 3.01 (s, 6H), 1.85 – 1.68 (m, 4H), 1.68 – 1.50 (m, 5H), 1.31 (q, *J* = 7.3 Hz, 2H), 1.27 – 0.97 (m, 6H), 0.93 (t, *J* = 7.3 Hz, 3H). **^{13}C NMR** (101 MHz, dmso) δ 72.13, 63.01, 62.07, 50.55, 43.91, 29.03, 28.16, 26.95, 26.56, 26.26, 26.15, 24.12, 19.61, 13.96. LC-MS (ESI-TOF): m/z 242.00 ($C_{15}H_{32}NO^+$ calcd 242.25).

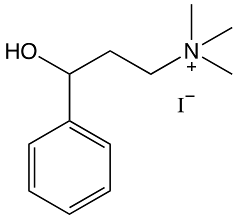

34Ba: Obtained in 63% yield as white solid. **^{1}H NMR** (400 MHz, DMSO-d_6) δ 7.45 – 7.32 (m, 4H), 7.32 – 7.22 (m, 1H), 5.58 (dd, *J* = 4.4, 0.6 Hz, 1H), 4.63 (dt, *J* = 8.5, 4.4 Hz, 1H), 3.52 – 3.33 (m, 2H), 3.06 (s, 9H), 2.09 – 1.93 (m, 2H). **^{13}C NMR** (101 MHz, dmso) δ 145.39, 128.62, 127.60, 126.11, 70.11, 63.96, 52.76, 52.72, 52.68, 32.45. LC-MS (ESI-TOF): m/z 194.30 ($C_{12}H_{20}NO^+$ calcd 194.15).

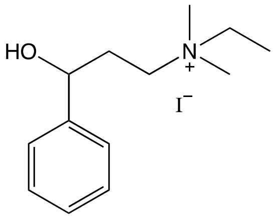

34Bb: Obtained in 54% yield as colorless semisolid. **^{1}H NMR** (400 MHz, DMSO-d_6) δ 7.49 – 7.31 (m, 4H), 7.31 – 7.23 (m, 1H), 5.57 (d, *J* = 4.4 Hz, 1H), 4.64 (dt, *J* = 8.4, 4.4 Hz, 1H), 3.44 – 3.25 (m, 4H), 2.99 (s, 6H), 2.08 – 1.87 (m, 2H), 1.29 – 1.13 (m, 3H). **^{13}C NMR** (101 MHz, dmso) δ 145.38, 128.60, 127.59, 126.11, 70.04, 60.81, 58.80, 50.12, 50.08, 32.04, 8.22. LC-MS (ESI-TOF): m/z 208.30 ($C_{13}H_{22}NO^+$ calcd 208.17).

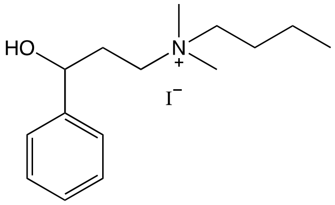

34Bc: Obtained in 75% yield as colorless semisolid. **^{1}H NMR** (400 MHz, DMSO-d_6) δ 7.47 – 7.30 (m, 4H), 7.31 – 7.23 (m, 1H), 5.57 (d, *J* = 4.4 Hz, 1H), 4.63 (dt, *J* = 8.5, 4.5 Hz, 1H), 3.43 – 3.33 (m, 2H), 3.29 – 3.18 (m, 2H), 3.01 (s, 6H), 1.99 (ddt, *J* = 14.0, 9.5, 5.7 Hz, 2H), 1.59 (p, *J* = 7.6 Hz, 2H), 1.28 (h, *J* = 7.4 Hz, 2H), 0.92 (t, *J* = 7.3 Hz, 3H). **^{13}C NMR** (101 MHz, dmso) δ 145.35, 128.60, 127.59, 126.13, 70.04, 63.03, 61.38, 50.65, 32.06, 24.08, 19.58, 13.94. LC-MS (ESI-TOF): m/z 236.30 ($C_{15}H_{26}NO^+$ calcd 236.20).

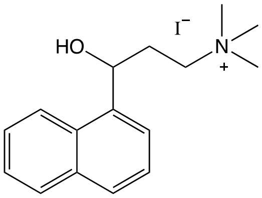

34Ca: Obtained in 86% yield as off white foamy solid. **^{1}H NMR** (400 MHz, DMSO-d_6) δ 8.20 (d, *J* = 8.4 Hz, 1H), 8.00 – 7.93 (m, 1H), 7.87 (d, *J* = 8.3 Hz, 1H), 7.76 – 7.69 (m, 1H), 7.63 – 7.46 (m, 3H), 5.72 (dd, *J* = 4.4, 0.8 Hz, 1H), 5.40 (dt, *J* = 8.2, 3.7 Hz, 1H), 3.69 (td, *J* = 12.3, 4.6 Hz, 1H), 3.58 (td, *J* = 12.3, 5.0 Hz, 1H), 3.07 (s, 9H), 2.27 – 2.14 (m, 1H), 2.08 (tdd, *J* = 12.9, 9.0, 4.9 Hz, 1H). **^{13}C NMR** (101 MHz, dmso) δ 140.59, 133.27, 129.62, 128.68, 127.50, 126.09, 125.52, 125.39, 123.11, 122.88, 66.50, 63.72, 52.23, 31.46. LC-MS (ESI-TOF): m/z 244.30 ($C_{16}H_{22}NO^+$ calcd 244.17).

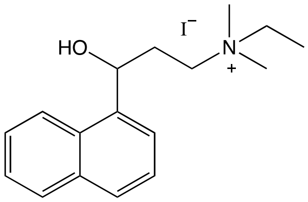

34Cb: Obtained in 83% yield as off white foam. **^{1}H NMR** (400 MHz, DMSO-d_6) δ 8.19 (dt, *J* = 8.5, 1.1 Hz, 1H), 8.00 – 7.93 (m, 1H), 7.86 (dt, *J* = 8.2, 0.9 Hz, 1H), 7.72 (dt, *J* = 7.0, 1.0 Hz, 1H), 7.62 – 7.48 (m, 3H), 5.73 (dd, *J* = 4.4, 0.7 Hz, 1H), 5.40 (dt, *J* = 8.1, 3.6 Hz, 1H), 3.62 (td, *J* = 12.5, 4.7 Hz, 1H), 3.50 (td, *J* = 12.4, 4.8 Hz, 1H), 3.33 (d, *J* = 7.6 Hz, 2H), 2.99 (s, 6H), 2.14 (td, *J* = 10.3, 8.6, 3.9 Hz, 1H), 2.04 (tdd, *J* = 13.0, 9.1, 4.8 Hz, 1H), 1.23 (t, *J* = 7.3 Hz, 3H). **^{13}C**

NMR (101 MHz, dmso) δ 141.08, 133.75, 130.09, 129.17, 127.98, 126.55, 126.00, 125.86, 123.54, 123.34, 66.92, 61.05, 58.94, 50.14, 49.97, 31.56, 8.21. LC-MS (ESI-TOF): m/z 258.30 ($C_{17}H_{24}NO^+$ calcd 258.19).

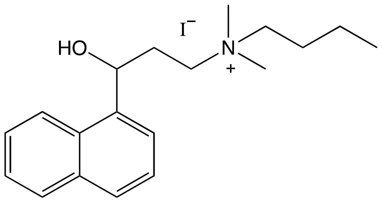

34Cc: Obtained in 60% yield as off white foam. **^{1}H NMR** (400 MHz, DMSO-d_6) δ 8.26 – 8.16 (m, 1H), 7.97 (dd, *J* = 7.9, 1.7 Hz, 1H), 7.87 (d, *J* = 8.3 Hz, 1H), 7.77 – 7.69 (m, 1H), 7.63 – 7.47 (m, 4H), 5.72 (s, 1H), 5.41 (dt, *J* = 8.1, 3.7 Hz, 1H), 3.64 (td, *J* = 12.4, 4.6 Hz, 1H), 3.50 (td, *J* = 12.5, 4.7 Hz, 1H), 3.30 – 3.17 (m, 2H), 3.01 (d, *J* = 2.2 Hz, 6H), 2.15 (ddt, *J* = 12.8, 7.9, 3.9 Hz, 1H), 2.05 (tdd, *J* = 13.2, 8.8, 4.7 Hz, 1H), 1.69 – 1.52 (m, 2H), 1.25 (h, *J* = 7.3 Hz, 2H), 0.90 (t, *J* = 7.3 Hz, 3H). **^{13}C NMR** (101 MHz, dmso) δ 141.00, 133.76, 130.08, 129.17, 127.98, 126.51, 126.01, 125.86, 123.57, 123.37, 66.97, 63.07, 61.47, 50.79, 50.61, 31.54, 24.11, 19.56, 13.93. LC-MS (ESI-TOF): m/z 286.30 ($C_{19}H_{28}NO^+$ calcd 286.22).

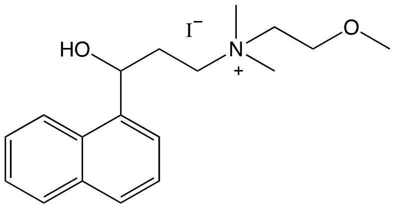

34Cd: Obtained in 65% yield as pale yellow semisolid. **^{1}H NMR** (400 MHz, DMSO-d_6) δ 8.22 – 8.14 (m, 1H), 7.99 – 7.94 (m, 1H), 7.86 (d, *J* = 8.1 Hz, 1H), 7.72 (dt, *J* = 7.0, 1.0 Hz, 1H), 7.61 – 7.49 (m, 3H), 5.72 (dd, *J* = 4.4, 0.7 Hz, 1H), 5.39 (dt, *J* = 8.3, 3.7 Hz, 1H), 3.80 – 3.49 (m, 6H), 3.28 (s, 3H), 3.08 (s, 3H), 3.06 (s, 3H), 2.19 (t, *J* = 12.6 Hz, 1H), 2.08 (ddd, *J* = 17.7, 12.5, 6.4 Hz, 1H). **^{13}C NMR** (101 MHz, dmso) δ 141.11, 133.74, 130.10, 129.14, 127.95, 126.50, 125.98, 125.85, 123.57, 123.36, 67.08, 65.80, 62.77, 58.55, 51.40, 40.23, 31.67. LC-MS (ESI-TOF): m/z 288.30 ($C_{18}H_{26}NO_2^+$ calcd 288.20).

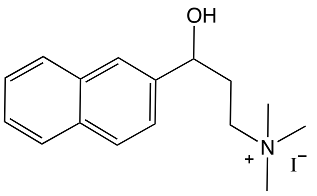

34Da: Obtained in 66% yield as white solid. **^{1}H NMR** (400 MHz, DMSO-d_6) δ 7.97 – 7.85 (m, 4H), 7.57 (dd, *J* = 8.5, 1.7 Hz, 1H), 7.55 – 7.46 (m, 2H), 5.73 (d, *J* = 4.3 Hz, 1H), 4.82 (dt, *J* = 8.3, 4.4 Hz, 1H), 3.45 (ddt, *J* = 14.6, 11.3, 7.0 Hz, 2H), 3.07 (s, 9H), 2.**24 – 2.01 (m, 2H). ^{13}C NMR** (101 MHz, dmso) δ 142.80, 133.22, 132.83, 128.26, 128.18, 127.96, 126.61, 126.19, 124.74, 124.43, 70.17, 63.83, 52.73, 32.23. LC-MS (ESI-TOF): m/z 244.30 ($C_{16}H_{22}NO^+$ calcd 244.17).

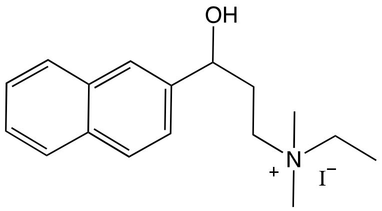

34Db: Obtained in 83% yield as yellow semisolid. **^{1}H NMR** (400 MHz, DMSO-*d*6) δ 8.01 – 7.83 (m, 4H), 7.57 (dd, *J* = 8.5, 1.7 Hz, 1H), 7.55 – 7.44 (m, 2H), 5.73 (d, *J* = 4.4 Hz, 1H), 4.82 (dt, *J* = 8.4, 4.3 Hz, 1H), 3.46 – 3.32 (m, 4H), 2.99 (s, 6H), 2.18 – 1.97 (m, 2H), 1.21 (t, *J* = 7.3 Hz, 3H). **^{13}C NMR** (101 MHz, dmso) δ 142.79, 133.21, 132.82, 128.25, 128.18, 127.97, 126.61, 126.19, 124.74, 124.43, 70.12, 60.73, 58.76, 50.14, 31.81, 8.22. LC-MS (ESI-TOF): m/z 258.30 ($C_{17}H_{24}NO^{+}$ calcd 258.19).

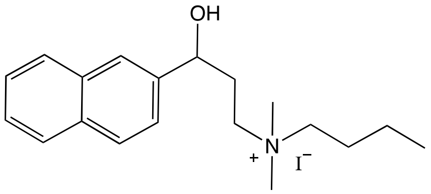

34Dc: Obtained in 89% yield as colorless glassy material. **^{1}H NMR** (400 MHz, DMSO-*d*6) δ 7.96 – 7.85 (m, 4H), 7.57 (dd, *J* = 8.5, 1.7 Hz, 1H), 7.55 – 7.46 (m, 2H), 5.73 (d, *J* = 4.3 Hz, 1H), 4.82 (dt, *J* = 8.4, 4.3 Hz, 1H), 3.47 – 3.35 (m, 2H), 3.30 – 3.19 (m, 2H), 3.01 (s, 6H), 2.10 (dh, *J* = 13.0, 7.1 Hz, 2H), 1.67 – 1.51 (m, 2H), 1.28 (h, *J* = 7.4 Hz, 2H), 0.90 (t, *J* = 7.3 Hz, 3H). **^{13}C NMR** (101 MHz, dmso) δ 142.74, 133.21, 132.83, 128.25, 128.18, 127.97, 126.62, 126.20, 124.74, 124.46, 70.12, 62.99, 61.27, 50.67, 31.82, 24.09, 19.58, 13.92. LC-MS (ESI-TOF): m/z 286.30 ($C_{19}H_{28}NO^{+}$ calcd 286.22).

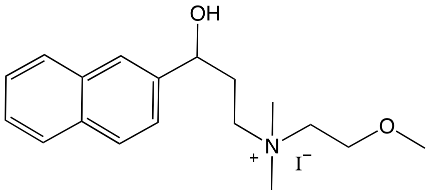

34Dd: Obtained in 68% yield as pale yellow semisolid. **^{1}H NMR** (400 MHz, DMSO-*d*6) δ 7.98 – 7.84 (m, 4H), 7.56 (dd, *J* = 8.5, 1.7 Hz, 1H), 7.55 – 7.44 (m, 2H), 5.73 (d, *J* = 4.3 Hz, 1H), 4.81 (dt, *J* = 8.4, 4.4 Hz, 1H), 3.72 (s, 2H), 3.62 – 3.42 (m, 4H), 3.26 (s, 3H), 3.07 (s, 3H), 3.07 (s, 3H), 2.22 – 2.03 (m, 2H). **^{13}C NMR** (101 MHz, dmso) δ 142.83, 133.21, 132.82, 128.23, 128.17, 127.96, 126.60, 126.18, 124.76, 124.43, 70.26, 65.82, 62.66, 62.53, 58.53, 51.35, 31.96. LC-MS (ESI-TOF): m/z 288.30 ($C_{18}H_{26}NO_2^{+}$ calcd 288.20).

1.4. Synthesis of Sulfone derivative 35

Synthesis of S4: To a stirred solution of phenyl vinyl sulfone **S3** (0.977 g, 5.81 mmol) in ethanol (10 mL) at room temperature, was added a 2M solution of dimethyl amine in methanol (0.739 g, 8.2 mL, 16.4 mmol) over 10 min. The mixture was stirred at room temperature for 30 min. The reaction was monitored by TLC. After completion the reaction mixture was concentrated under vacuum to dryness to afford desired product **S4** as colorless oil (1.2 g, 97%). **^{1}H NMR** (400 MHz, Chloroform-*d*) δ 7.95 – 7.88 (m, 2H),

7.70 – 7.62 (m, 1H), 7.61 – 7.53 (m, 2H), 3.33 – 3.19 (m, 2H), 2.77 – 2.64 (m, 2H), 2.17 (s, 6H). **^{13}C NMR** (101 MHz, cdcl$_3$) δ 133.70, 129.22, 127.99, 109.99, 54.02, 52.22, 44.99. LC-MS (ESI-TOF): m/z 214.20 ([C10H15NO2S + H]$^+$ calcd 214.08).

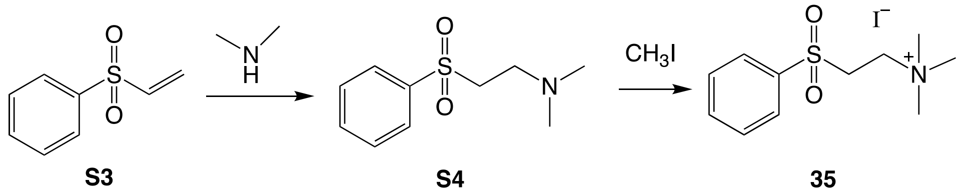

Scheme S2

Synthesis of 35: To a stirred solution of N,N-dimethyl-2-(phenylsulfonyl)ethan-1-amine **S4** (0.34 g, 1.6 mmol) in ethanol (5 mL) at room temperature, was added methyl iodide (0.34 g, 2.4 mmol) and reaction mixture was stirred for 18 h. The solvent was removed using nitrogen flow to afford semisolid, which was taken in diethyl ether (~25 mL) and stirred for 30 min. The solid was collected by filtration and washed with copious amounts of diethyl ether and dried in a high vacuum to afford the desired compound **35** as off white solid (0.45 g, 79%). **^{1}H NMR** (400 MHz, DMSO-d_6) δ 8.00 – 7.90 (m, 2H), 7.87 – 7.78 (m, 1H), 7.76 – 7.66 (m, 2H), 4.12 – 3.96 (m, 2H), 3.74 – 3.60 (m, 2H), 3.07 (s, 9H). **^{13}C NMR** (101 MHz, dmso) δ 138.80, 135.04, 130.20, 128.24, 58.26, 52.98, 48.94. LC-MS (ESI-TOF): m/z 228.20 ($C_{11}H_{18}NO_2S^+$ calcd 228.11).

1.5. Synthesis of elongated linker chain length α-NETA analogues 36A-B

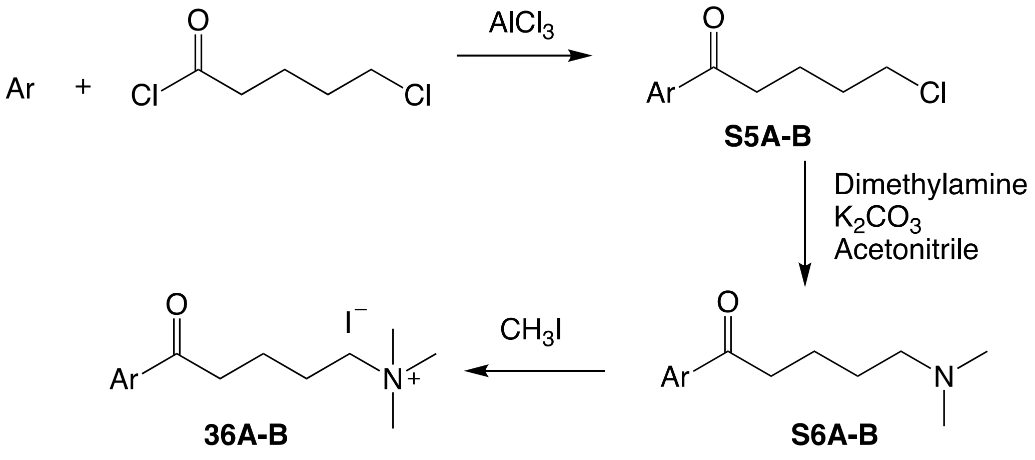

A = Ph
B = β-Napthyl

Scheme S3

Synthesis of S5A-B: Under a nitrogen atmosphere, $AlCl_3$ (1.1 eq, 43.0 mmol) was taken in dichloromethane (25 mL) in a single-neck round-bottom flask. The mixture was cooled to 0 – 5 °C and then 5-chloropentanoyl chloride (1.0 eq, 39.0 mmol) was added dropwise while keeping the reaction temperature below 5 °C. To the reaction mixture a solution of either benzene or naphthalene in dichloromethane (25 mL) was then added dropwise over 30 min, giving yellow solution. The reaction mixture was then stirred for 2h at room temperature and poured on crushed ice. Stirring was continued for 30 min then organic layer was separated. Aqueous layer was extracted again with dichloromethane (50 mL). Combined organic layer was washed with water, brine, dried over Na_2SO_4, filtered and evaporated to dryness to afford the crude compound that was further purified on silica gel column with 0-40% DCM in hexane as the eluent to obtain desired product **S5** as an off white solid.

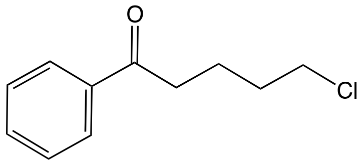

S5A: Obtained in 83% yield as off white solid. **^{1}H NMR** (400 MHz, Chloroform-*d*) δ 8.00 – 7.90 (m, 2H), 7.61 – 7.52 (m, 1H), 7.50 – 7.40 (m, 2H), 3.62 – 3.53 (m, 2H), 3.01 (t, *J* = 6.9 Hz, 2H), 1.97 – 1.81 (m, 4H). **^{13}C NMR** (101 MHz, cdcl$_3$) δ 199.56, 136.83, 133.05, 128.60, 127.98, 127.97, 44.70, 37.53, 32.04, 21.49.

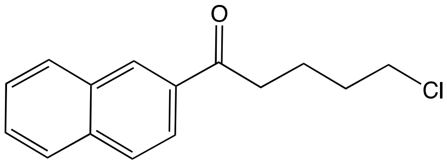

S5B: Obtained in 42% yield as pale yellow solid. **^{1}H NMR** (400 MHz, Chloroform-*d*) δ 8.59 (dd, *J* = 8.6, 0.8 Hz, 1H), 8.01 – 7.94 (m, 1H), 7.91 – 7.81 (m, 2H), 7.63 – 7.44 (m, 3H), 3.59 (t, *J* = 6.2 Hz, 2H), 3.08 (t, *J* = 6.9 Hz, 2H), 2.01 – 1.84 (m, 4H). **^{13}C NMR** (101 MHz, cdcl$_3$) δ 203.91, 135.93, 133.97, 132.58, 130.10, 128.45, 127.91, 127.37, 126.47, 125.71, 124.37, 44.72, 41.09, 32.06, 21.95. LC-MS (ESI-TOF): m/z 247.10 ($[C_{15}H_{15}ClO + H]^+$ calcd 247.08).

Synthesis of S6A-B:

In a two-neck round-bottom flask, fitted with a reflux condenser was suspended **S5** (1 eq, 10.2 mmol) in acetonitrile (50 mL). Then dimethyl amine HCl salt (1.2 eq, 12.2 mmol) and K_2CO_3 (2.4 eq, 25 mmol) were added. The reaction mixture was stirred at refluxed for 24 h. The reaction

was monitored by TLC for the absence of the starting material. After cooling to ambient temperature, it was poured into ice-cold water (~150 mL) and stirred for 10 min then extracted with dichloromethane (2 X 100 mL). combined organic layer was washed with water, brine, dried over Na_2SO_4 and filtered. The organic layer was evaporated to dryness and the residue was purified by column chromatography by using 0-5% Methanol in dichloromethane to afford the desired compound **S6** as a brown oil.

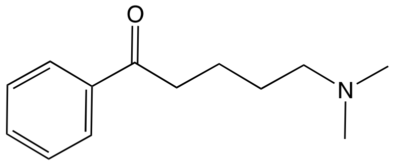

S6A: Obtained in 86% yield as brown oil. **^{1}H NMR** (400 MHz, Chloroform-*d*) δ 7.96 – 7.88 (m, 2H), 7.54 – 7.47 (m, 1H), 7.46 – 7.37 (m, 2H), 3.01 – 2.89 (m, 2H), 2.28 (s, 2H), 2.18 (s, 6H), 1.73 (ddt, *J* = 8.5, 7.8, 7.1 Hz, 2H), 1.58 – 1.45 (m, 2H). **^{13}C NMR** (101 MHz, cdcl$_3$) δ 200.09, 136.99, 132.83, 128.49, 127.98, 59.52, 45.49, 38.35, 27.39, 22.16. LC-MS (ESI-TOF): m/z 206.30 ([$C_{13}H_{19}NO$ + H]$^+$ calcd 206.15).

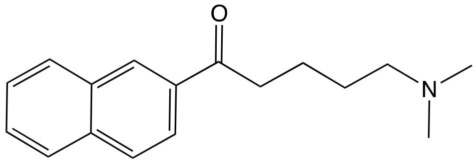

S6B: Obtained in 78% yield as brown semisolid. **^{1}H NMR** (400 MHz, Chloroform-*d*) δ 8.59 – 8.48 (m, 1H), 7.96 (dt, *J* = 7.9, 1.0 Hz, 1H), 7.89 – 7.78 (m, 2H), 7.63 – 7.41 (m, 3H), 3.07 (t, *J* = 7.3 Hz, 2H), 2.34 – 2.25 (m, 2H), 2.21 (s, 6H), 1.87 – 1.74 (m, 2H), 1.64 – 1.51 (m, 2H). **^{13}C NMR** (101 MHz, cdcl$_3$) δ 204.67, 136.31, 133.94, 132.31, 130.10, 128.37, 127.77, 127.16, 126.38, 125.74, 124.34, 59.57, 45.53, 42.05, 27.43, 22.60. LC-MS (ESI-TOF): m/z 256.30 ([$C_{17}H_{21}NO$ + H]$^+$ calcd 256.16).

Synthesis of 36A-B:

To a stirred solution of **S6** (1.0 eq, 1.1 mmol) in ethanol (5 mL) at room temperature, was added methyl iodide (1.5 eq, 1.7 mmol) and stirred for 24 h, during which time the a white solid precipitated. The solid was collected by filtration and washed with copious amounts of ethanol followed by diethyl ether. The solid was further purified by stirring them in mixture of acetonitrile and diethyl ether for overnight.

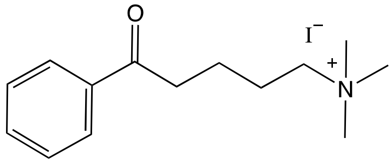

36A: Obtained in 80% yield as off white solid. **^{1}H NMR** (400 MHz, DMSO-d_6) δ 8.00 – 7.91 (m, 2H), 7.67 – 7.59 (m, 1H), 7.57 – 7.47 (m, 2H), 3.40 – 3.31 (m, 2H), 3.12 (t, *J* = 7.0 Hz, 2H), 3.04 (s, 9H), 1.82 – 1.68 (m, 2H), 1.68 – 1.55 (m, 2H). **^{13}C NMR** (101 MHz, dmso) δ 199.82, 136.98, 133.66, 129.18, 128.31, 65.54, 52.65 (t, *J* = 4 Hz), 37.59, 22.10, 20.77. LC-MS (ESI-TOF): m/z 220.30 ($C_{14}H_{22}NO^+$ calcd 220.17).

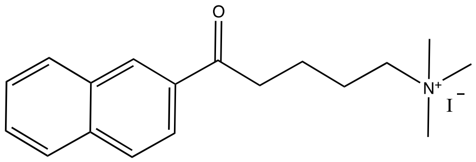

36B: Obtained in 78% yield as off white solid. **^{1}H NMR** (400 MHz, DMSO-d_6) δ 8.51 – 8.42 (m, 1H), 8.15 (d, *J* = 8.2 Hz, 1H), 8.12 – 8.06 (m, 1H), 8.06 – 7.98 (m, 1H), 7.69 – 7.50 (m, 3H), 3.43 – 3.33 (m, 2H), 3.22 (t, *J* = 7.0 Hz, 2H), 3.07 (s, 9H), 1.81 (p, *J* = 8.0, 7.5 Hz, 2H), 1.71 (p, *J* = 7.1 Hz, 2H). **^{13}C NMR** (101 MHz, dmso) δ 204.17, 135.92, 133.93, 132.82, 129.78, 128.98, 128.35, 128.19, 126.87, 125.71, 125.29, 65.54, 52.65 (t, *J* = 4 hz), 41.08, 22.09, 21.17. LC-MS (ESI-TOF): m/z 270.30 ($C_{18}H_{24}NO^+$ calcd 270.19).
